# Environmental and health impacts of pharmaceuticals in radish crops irrigated with reclaimed water

**DOI:** 10.1007/s11356-025-36864-9

**Published:** 2025-08-27

**Authors:** Lamia Benelhadj-Djelloul Guetni, Pedro Antonio Nortes Tortosa, Laura Ponce Robles

**Affiliations:** Centro de Edafologia y Biologia Aplicada del Segura, CEBAS-CSIC, Espinardo, Murcia, Spain

**Keywords:** Bioconcentration and translocation, Crop irrigation, Environmental risks, Health risks, Pharmaceutical uptake, Reclaimed water, Soil accumulation and leaching

## Abstract

The use of reclaimed water for crop irrigation can be an important route for pharmaceutical introduction into the food chain. This work aims to investigate the crop uptake, accumulation and translocation of 11 representative pharmaceuticals in radish crop through two realistic agricultural growing conditions (highland and fall radish). For this, radish crops were irrigated with three reclaimed water qualities: (i) WWTP effluent (named real effluent, RE), (ii) RE fortified with 5 µg/L of pharmaceuticals (named higher concentration, HC) and (iii) RE fortified with a concentration of pharmaceuticals 10 times lower than HC (0.5 µg/L, named medium concentration, MC). Results revealed that the pharmaceutical concentration in irrigation water does not play a role in radish quality. However, phytotoxic effects could appear at concentrations above 100 µg/L. A clear relation between irrigation water quality and uptake was obtained (RE < MC < HC). Pharmaceutical concentrations in radish leaves were higher than in roots in all cases. However, the total distribution percentages in radish tissues and soil were directly dependent on the environmental growing conditions (mainly attributed to evapotranspiration rates, ET). Octanol–water partition coefficient (log *K*_ow_) of each pharmaceutical was a critical factor in radish uptake, accumulation and translocation (optimal range 1–4), while organic carbon to water partition coefficient (*K*_oc_) played a key role in soil accumulation rates. The concentration levels of pharmaceuticals detected in the edible part of radish did not pose a risk to human health via dietary intake. However, irrigation with reclaimed water containing pharmaceuticals (mainly caffeine (CAF), carbamazepine (CBZ) and sulfamethoxazole (SMX)) could have a significant environmental impact for aquatic and terrestrial organisms. The link between risk assessment and future prospects was also discussed.

**Graphical Abstract:**

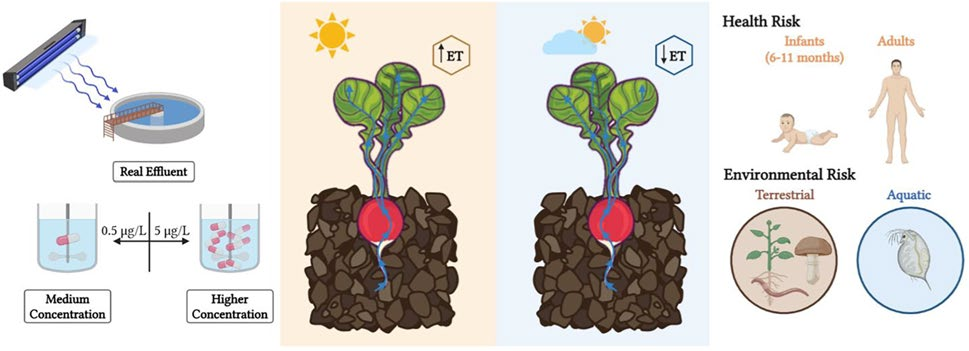

**Supplementary Information:**

The online version contains supplementary material available at 10.1007/s11356-025-36864-9.

## Introduction

Water scarcity, together with increasing food needs, is the world’s greatest challenge currently facing humanity (Fito and Van Hulle 2021). The agricultural sector is both a cause and a hazard of scarcity problems, accounting for almost 70% of all water withdrawals and up to 95% in some developing countries (Winpenny et al. 2010). In particular, the water demand for irrigation is estimated to be 2.9 thousand km^3^ in 2050 (FAO 2015). This translates into the need for an increase in irrigation water of about 15%, a value that according to FAO (2018) could not be reached (as it is estimated that the maximum increase is 10%).

Treatment and reuse of wastewater from wastewater treatment plants (WWTP) for crop irrigation is considered the main option to combat water scarcity and contribute significantly to sustainable development (Salgot and Folch 2018). In the European Union, the concept of reuse is widespread and is part of various initiatives and guidelines, such as the European Green Deal (Plan New Circular Economy Action 2020) or the Regulation (EU) 2020/741 on minimum requirements for water quality. Water reuse also contributes to the Farm to Fork Strategy’s goal of reducing the environmental footprint of the EU food system and strengthening its resilience by providing an alternative, more reliable water source for irrigation (Wesseler 2022).

Although more than 40,000 million m^3^ of wastewater is treated in the EU every year, only 964 million m^3^/year (2.4%) of this treated wastewater is reused (Berbel et al. 2023). Mediterranean countries (with a large water deficit) such as Greece, Italy or Spain only reuse between 5 and 12% of their WWTP effluents (Hristov et al. 2021), which indicates both an opportunity and a need for reuse. This is due to limiting factors such as technical feasibility (need for appropriate treatment and management strategies), economic factors (the cost of distributing water from WWTP to agricultural areas), social factors (acceptance by farmers and consumers) and regulatory considerations (compliance with regulations and guidelines) (Shoushtarian and Negahban-Azar 2020).

Agricultural irrigation with WWTP effluents (also known as reclaimed water) has both negative and positive outcomes. Specifically, reclaimed water could supply a large part of the crop nutrient requirements (Mohammad Rusan et al. 2007), reducing the need for fertilizers (Bedbabis et al. 2010), assuming a direct positive impact on farmers and the environment. Other added benefits include the conservation of groundwater reservoirs, pollution control and even cost savings from other more expensive alternative sources of supply such as desalination waters (Jaramillo and Restrepo 2017; Christou et al. 2024). However, reclaimed water is considered one of the main entrance pathways for a broad variety of organic micropollutants (named contaminants of emerging concern, CECs) into the soil–plant system, since these are not fully removed after conventional WWTP tertiary treatments (Rizzo et al. 2019). Among all CECs, pharmaceutical compounds are perhaps the most worrying, even at low concentrations (ng-mg/L), mainly due to medical advances, resulting in an increase in the number of pharmaceuticals prescribed (Garduño-Jiménez and Carter 2024; Sleight et al. 2023). Their presence in the environment has become a widespread problem, because they can also affect health, posing a new challenge for water authorities and new regulations. Although the recently published Regulation (EU) 2020/741 (Regulation 2020) does not include concentration limits for pharmaceuticals in irrigation water, these compounds are listed in Annex II, and their monitoring should be considered a quality requirement for a risk management plan. The dispersion of pharmaceuticals in crops could have far-reaching consequences, including adverse effects on soil microbial communities, the emergence of antimicrobial resistance, contamination of the food chain through crop uptake, negative impacts on crop yields (seed germination, biomass growth) and potential degradation of soil quality owing to detrimental effects on soil organisms (Pinto et al. 2022; Sleight et al. 2023). In addition, their presence and persistence in agricultural ecosystems could result in undesired short long-term toxic effects for crops, soil and human health. However, the impact generated is not entirely clear and depends on numerous factors such as crop type, environmental factors, physicochemical properties of pharmaceuticals and soils and plant physiology (Christou et al. 2019). Other apparent dilemmas for assessing the impact of the use of reclaimed water in agriculture lie in the large number of pharmaceuticals present in the reclaimed water, the different concentrations at which they may be found and their potential synergistic effects. Under this perspective, there is a strong need to increase the knowledge on the behaviour of pharmaceuticals both in soil–plant systems (such as uptake levels, translocation or exposure), in order to reach an adequate risk assessment of the health and environmental impact of the irrigation of crops with reclaimed water under real agricultural conditions.

Root vegetables (such as radish) are the crops that may represent the worst-case scenario of direct contact between reclaimed water, soil and the consumed crop, due to different factors: (i) their high uptake capacity (Christou et al. 2019); (ii) its common edible part (roots) is in direct contact with the bioavailable fraction of pharmaceuticals present in soil-pore water, allowing it to penetrate into their tissues; (iii) its consumption is mainly raw, especially as a crunchy salad; (iv) the consumption of radish roots, leaves and sprouts has increased in recent years due to their high nutritional value and potential health benefits (good source of calcium, magnesium, copper, manganese, potassium, vitamin B6, vitamin C and folate Gupta et al. 2003; Gamba et al. 2021)). Spain is one of the leading European producers of fresh vegetables including radish (23.7% of the EU’s harvested production in 2022), followed by Italy (20.8%), and one of the largest exporters of radish worldwide (European Commission 2022). Spain was one of the main exporting countries in 2023 (TRIDGE 2024). Therefore, this study aims to evaluate the impact on the soil–plant atmosphere continuum (SPAC) (fate, uptake, accumulation and translocation) of 11 pharmaceuticals in radish crops growing via reclaimed water under common commercially agricultural practices. The study determines the compounds with higher capacity in reaching radish tissues (roots and leaves) and their accumulation in two different soil depths. Finally, the potential human risks from consuming the edible part of radish and the potential environmental risks from soil accumulation are assessed.

## Materials and methods

### Selected pharmaceuticals

Eleven pharmaceuticals from different therapeutic groups (including five non-steroidal anti-inflammatory drugs (NSAIDs), two antibiotics, two anticonvulsants, one beta-blocker and one stimulant) were selected as a case study due to their frequent identification in WWTP effluents and influents worldwide according to Tran et al. (2018). Analytical standards of all target compounds were purchased from Sigma-Aldrich® with a purity higher than 99%. The selected pharmaceuticals along with their physicochemical properties are listed in Table 1.

**Table 1:** List of target compounds with their Chemical Abstracts Service (CAS) number, chemical formula, net charge, water solubility, octanol–water partition coefficients (log *K*_ow_), organic carbon to water partition coefficients (*K*_oc_) and acid dissociation constants (pK_a_)

Compound	Chemical formula	Ion charge	CAS number	Mw (g/mol)	Water solubility (mg/L)	Log *K*_ow_	*K*_oc_	pKa
Acetaminophen (ACT) NSAID	C_8_H_9_NO_2_	Neutral	103–90-2	151.16	14,000 (at 25 °C)	0.46	21	9.38
Atenolol (ATE) Beta-blocker	C_14_H_22_N_2_O_3_	Cationic	29122–68-7	266.34	13,300 (at 25 °C)	0.16	148^1^	9.6
Caffeine (CAF) Stimulant	C_8_H_10_N_4_O_2_	Neutral	58–08-2	194.19	21,600 (at 25 °C)	− 0.07	741–7762	14.0
Carbamazepine (CBZ) Anticonvulsant	C_15_H_12_N_2_O	Neutral	298–46-4	236.27	18 (at 25 °C)	2.45	510	13.9
Diclofenac (DCF) NSAID	C_14_H_11_Cl_2_NO_2_	Anionic	15307–86-5	296.15	2.37 (at 25 °C)	4.51	245	4.15
Erythromycin (ERY) Antibiotic	C_37_H_67_NO_13_	Neutral	114–07-8	733.90	2000	3.06	570	8.9
Indomethacin (IND) NSAID	C_19_H_16_ClNO_4_	Anionic	53–86-1	357.8	0.937	0.91	1400	4.50
Ketoprofen (KTP) NSAID	C_16_H_14_O_3_	Anionic	22071–15-4	254.29	51 (at 22 °C)	3.12^2^	-	4.45
Naproxen (NPX) NSAID	C_14_H_14_O_3_	Anionic	22204–53-1	230.26	15.9 (at 25 °C)	3.18	330	4.15
Sulfamethoxazole (SMX) Antibiotic	C_10_H_11_N_3_O_3_S	Anionic	723–46-6	253.28	610 (at 37 °C)	0.89	72	1.6/5.7
Sulpiride (SUL) Anticonvulsant	C_15_H_23_N_3_O_4_S	Cationic	15676–16-1	341.4	51.2	-	-	9.12

Data from PubChem (2024)

*NSAID* non-steroidal anti-inflammatory drug

^1^Bueno, et al., (2022)

^2^Wu et al. (2018)

### Irrigation water source

Reclaimed water used for radish crop irrigation was obtained from a conventional WWTP located in Balsicas (Murcia, Spain, latitude 37° 47′ 48″ N, longitude 0° 57′ 36″ W). This plant has a maximum treatment capacity of 2,007,500 m^3^/year. The WWTP design consists of a primary physicochemical treatment, a double-stage activated sludge with prolonged aeration and a tertiary treatment based on UV irradiation. Effluents from this WWTP are commonly used for agricultural irrigation (mainly leafy and root vegetables), as it is located in one of the most important agricultural areas of the Murcia Region. Physicochemical properties of selected WWTP effluents and pharmaceutical concentration ranges are shown in Tables S1 and S2.

Three irrigation water qualities in terms of pharmaceutical concentration were used for irrigation purposes: (i) WWTP effluent (named real effluent, RE), (ii) RE fortified with 5 µg/L of pharmaceuticals (named higher concentration, HC) and (iii) RE fortified with a concentration of pharmaceuticals 10 times lower than the HC (0.5 µg/L, named medium concentration, MC). Selected concentration values are in line with concentration minimum and maximum ranges for the selected compounds in worldwide WWTP effluents according to Tran et al. (2018).

The stock solutions containing all pharmaceutical compounds (fortified experiments) were prepared in deionized water weekly and kept refrigerated (4 °C) until use. The concentration of these solutions was verified in all cases by means of its analysis by quadrupole time-of-flight mass spectrometry (HR-QTOF-MS).

### Experimental design and sampling strategy

Round radish (*Raphanus raphanistrum*, red-white tip), purchased by Rocalba, S.A. (Girona, Spain), was grown from seeds under common agricultural practices in an experimental 680 m^2^ greenhouse belonging to a research centre of the Spanish National Research Council (CSIC), Segura Centre of Soil Science and Applied Biology (CEBAS). The greenhouse was located next to the selected WWTP (see the “Irrigation water source” section), allowing direct crop irrigation with reclaimed water. Two different growth cycles were evaluated using the same agricultural soil, simulating common harvest practices and periods according to Qiao et al. (2023): (a) first crop in October 2020 (highland radish) and (b) second crop in December 2020 (fall radish).

Experiments were carried out in a real sandy loam soil (4.2% sand, 64.8% silt and 30.9% clay), with a density of 8 plants/m^2^ (for more information, see Table S3). The total area for each growing scenario was 36 m^2^, which was divided into nine plots of 4 m^2^ each (three plots for each water quality, RE, MC and HC), and the crop distribution was done randomly to prevent potential effects of the spatial variation inside the greenhouse (for more information, see Fig. S1). The total irrigation water volume applied through a drip irrigation system (flow of 2.2 L/h) was 2.87 m^3^ and 2.77 m^3^ for the first and second crop, respectively. All experiments were conducted under natural light conditions, and weather conditions (air temperature (*T*^a^), net radiation (*R*_n_) and relative humidity (RH)) were recorded in continuous mode during the experimental time by CEBAS-CSIC’s weather station installed inside the greenhouse. In the first crop, *T*^a^ and *R*_n_ were on average 7.1 °C and 138.53 MJ/m^2^-day higher and RH 4% lower than in the second crop (for more information, see Table S4). In both radish growth scenarios, commercial practices for fertirrigation and pest management were followed.

Radishes’ tissues (including roots and leaves) from both crops were freshly harvested at the end of the growing cycle (21 days for first crop and 30 days for second crop, corresponding to radish maturity stage). Five representative radishes of each plot from each replicated water quality treatment (RE, MC and HC) were collected and combined as one single sample (*n*_total_ = 45). The roots and the leaves were separated from each radish. Then, samples were washed with tap water to remove soil particles adhered to the plant surface and to simulate the usual practice of vegetable producers or consumers before consumption. The samples were weighed before being stored in plastic bags and transported to the CEBAS-CSIC laboratory for analysis. All samples were stored at 4 °C and extracted within 1 day of collection.

Three soil replicates from the first crop (at the top 0–5 cm of the soil profile where radishes typically grow) and the second crop (0–5 cm and 25–30 cm) were analyzed with the aim of obtaining accurate information on potential effects that could occur in real soils due to pharmaceutical occurrence, accumulation and their impact on leaching. All soil samples were collected separately in a sample storage bag and stored at 4 °C until analysis.

### Analysis of pharmaceuticals in the soil–plant system

The quantification of selected pharmaceuticals in the soil–plant system was performed by an extraction step based on the QuEChERS methodology followed by a HR-QTOF-MS analysis, according to Ponce-Robles et al. (2022).

Prior to extraction, fresh radish tissues were crushed and homogenized, while soil samples were homogenized and oven dried at 40 °C until constant weight. In all cases, samples were extracted within 24 h of preparation.

For radish roots and leaves, 10 g of fresh sample was added to a 50-mL centrifuge tube containing 10 mL of acetonitrile (0.1% acetic acid). The samples were shaken for 30 s and left for 15 min. Then, a mixture of extraction salts (6 g MgSO_4_ and 1.5 g of NaOAc) was added, and the mixture was shaken vigorously for 10 min. For the clean-up step, 5 mL of the extract was transferred to a 15-mL centrifuge tube containing 750 mg of MgSO_4_ and 125 mg of C18. After that, the samples were vortexed for 30 s and centrifuged at 3500 rpm (5 min). Finally, an aliquot of 1 mL was evaporated to dryness under nitrogen stream and reconstituted with a mixture of MeOH:H_2_O (5:95) containing 0.1% acetic acid. In the case of soil samples, 10 mL of acetonitrile was added to 1 g of dry soil rehydrated with 5 mL of Milli-Q water, and a similar extraction procedure was performed. Carbamazepine-d10 was selected as the surrogate standard to check the extraction efficiency. For more information on the selected extraction procedure, see Fig. S2.

All extracts were analyzed using an ACQUITY UPLC I-Class System coupled with an HR-QTOF-MS maXis Series (Bruker Daltonik GmbH, Germany), equipped with an ACQUITY BEH C18 (100 mm × 2.1 mm, 1.7 µm) analytical column. For conditions, see general information S1.

#### Quality control and assurance

As quality control, a target method validation was carried out in the selected matrices (radish tissues and soil). Recovery rates were in all cases between 70 and 120%, with associated precision RSD < 20%, which are satisfactory mean recoveries according to Martínez-Piernas et al. (2019). Linear range and method quantification limits (MQLs) were also evaluated (see Table S5). Matrix-matched calibration curves prepared in sample extracts were used for the quantitative analysis. A standard mixture of selected pharmaceuticals was injected each day before analysis in order to check the functioning of the analytical column and the mass spectrometer. Blank samples (solvent) were also included during the daily work sequence. In accordance with the International System of Units, the obtained pharmaceutical concentrations for the vegetable tissue were presented as fresh weight (f.w.) for radish roots’ tissues, whereas for the soil, they were presented as dry weight (d.w.).

### Fruit quality and radish yield

Radish production (average weight per treatment (grammes)), diameter (centimetre) and moisture content (%)) was measured at the end of the growing cycle for each collected sample. The measurements were made in triplicate. Colour was measured in the inner part of sliced radish roots with a colorimeter (Konica Minolta CR-10; Konica Minolta Sensing, Japan), calibrated to a white reference plate. The colour space coordinates L*, a*, b* and chroma ((a*2 + b*2)1/2) were measured in each radish root (five repetition for each treatment) from three different positions around the equatorial zone to obtain the Hue° index following the equation: [H° = arctg (a*/b*)] (Andrés and Perla 2004; Saad et al. 2016). L* indicates lightness (whiteness or brightness/darkness), a* indicates chromaticity on a green (−) to red (+) axis and b* indicates chromaticity on a blue (−) to yellow (+) axis (CIE 1978).

### Bioconcentration factor and translocation factor

The bioconcentration factor in roots (RCF) and translocation factor (TF) were used to estimate, respectively, the uptake and translocation capacity of pharmaceuticals in radish tissues (Kim et al. 2023). The RCF is calculated as the ratio of each pharmaceutical in roots (edible crop part) in the growth medium (Eq. 1), while TF is calculated through leaf/root concentration ratio according to González García et al. (2018) (Eq. 2). An easy translocation from root to leaf is considered for TF ≥ 1 values.

1 $$RCF\, \left(L/kg\right)= \frac{concentration\, in\, roots\, (\mu g/kg) }{concentration\, in\, irrigation\, water \,(\mu g/L)}$$

2 $$TF= \frac{concentration\, in \,leaf }{concentration\, in \,roots}$$

### Statistical Analysis

A weighted analysis of variance (ANOVA), followed by Student’s *t*-test (*p* < 0.05), was used for assessing physicochemical differences among the experiments, according to Mira-García et al. (2024). Further, the data were analyzed using a two-way ANOVA with irrigation water qualities and the two different growing environments as the main factors. The statistical analyses were performed with SPSS statistical software (v. 25.0, IBM Corporation Armonk, NY, USA).

### Risk assessment

The potential health and ecotoxicological risks of the target pharmaceuticals in radish crops were assessed in order to know the short- and long-term impact generated by the presence of these compounds in agricultural environments irrigated with reclaimed water.

#### Health risk assessment

The human health risks associated with the consumption of the selected pharmaceuticals through intake of radish roots irrigated with reclaimed water were carried out by calculating the hazard quotient (HQ) in two different age groups: adults between 18 and 75 years and also in infants between 6 and 11 months in order to include the worst-case scenario due to the low body weight of this population group. HQ was calculated from the ratio of estimated dietary intake (EDI) to acceptable dietary intake (ADI), following Eq. 3:

3 $$HQ= \frac{EDI}{ADI}$$

where ADI was obtained from literature (for more information, see Table S7) and EDI was estimated following Eq. 4:

4 $$EDI\,\left(ng/kg-day\right)=\frac{concentration\,(ng/g)\times consumption\,(g/day)}{body\,weight\,(kg)}$$

For the EDI calculations, an average body weight for adults and infants was set at 71.8 kg and 9.1 kg, respectively, according to US Epa (1997). In addition, according to data from the Spanish Agency for Food Safety and Nutrition, the consumption of this tuber is 16.2 g/day for adults and 16.6 g/day for infants (AESAN 2014). Additionally, HQ associated with the intake of radish leaves was also estimated, due to the increase in their consumption in recent years (Lee et al. 2023). In the absence of data on the daily consumption of this part of radish, the estimated daily intake of radish roots was considered for the calculations.

Humans exposed to a given pharmaceutical are considered to be at no risk of adverse health effects when the HQ value for each chemical is less than 1 (Kumar and Xagoraraki 2010).

Additionally, the cumulative health hazard index (HI) was estimated using Eq. 5, according to Keerthanan et al. (2020) and Zhao et al. (2019), where *n* was the number of target pharmaceuticals selected in this study. The authors reported negligible human threat when HI < 0.01, moderate threat when 0.01 < HI < 0.05 and high threat when HI > 0.05.

5 $$HI={\sum }_{i}^{n}{RQ}_{i}$$

#### Ecotoxicological risk assessment

Ecotoxicological risk of selected CECs present in agricultural soils under reclaimed water irrigation practices was calculated as the ratio between the maximum environmental concentration of each pharmaceutical detected in soil (MEC_SOIL_) and the concentration below which no adverse effects are expected to occur (predicted no-effect concentrations; PNEC_SOIL_), following Eq. 6:

6 $$RQ=\frac{{MEC}_{SOIL}}{{PNEC}_{SOIL}}$$

7 $${PNEC}_{SOIL}=\frac{{L(E)C}_{50}}{AF}$$

where PNEC_SOIL_values was estimated by dividing short-term toxicity tests (L(E)C_50_) and an appropriate assessment factor (AF), which is an arbitrary value associated with the uncertainty in extrapolating from a limited number of laboratory species to complex ecosystems (Vazquez-Roig et al. 2012; Bouissou-Schurtz et al. 2014); EMA (2024), (see Eq. 7). L(E)C_50_ values correspond to the lowest toxicity data reported in ECOTOX database (ECOTOX 2024) for terrestrial organisms and AF is equal to 1000, as recommended in the (European Commission’s Technical Guidance on Risk Assessment 2003). To cover the food chain, RQ was calculated at three different trophic levels of the ecosystem: earthworms, plants and fungi. However, no data available for all selected compounds in terrestrial species. According to (European Commission’s Technical Guidance on Risk Assessment 2003), the risk assessment should also be performed on the basis of the result of aquatic toxicity data if toxicity test results are not available for more than one terrestrial organism. Thus, the equilibrium partitioning method was applied to identify the predicted no effect concentrations for soil organisms from the toxicity tests on aquatic organisms (Aydın et al. 2022); Pu et al. 2022; Mejías et al. 2024). In this case, PNEC_SOIL_ value was estimated from the soil-water distribution coefficient (k_d_) of each selected pharmaceutical multiplied by predicted no-effect concentrations in the aquatic environment (PNEC_WATER_) value (Eq. 8):

8 $${PNEC}_{SOIL}={k}_{d}\times {PNEC}_{WATER}$$

PNEC_WATER_ was calculated by dividing the lowest L(E)C50 value reported in the ECOTOX database for crustaceans, as representative species for ecotoxicological assessment of contaminants, sensitive to environmental changes and also reliable indicators of ecosystem health (Bertrand et al. 2018), by an assessment factor of 1000 according to the indications of the European Commission’s Technical Guidance on Risk Assessment (2003).

For the interpretation of the risk level, the criteria established by Mozas-Blanco et al. (2023) were followed: negligible risk (RQ < 0.1), low risk (0.1 < RQ < 1), moderate risk (1 < RQ < 10) and high risk (RQ > 10).

## Results and discussion

### Crop yield effects and status health from pharmaceutical exposure

Radishes grown in all experiments (three types of irrigation water (RE, MC and HC) and both environments (highland radish and fall radish)) were found to be generally healthy. No macroscopic phytotoxic symptoms of stress (such as chlorosis due nutrient deficiency) were observed. Colour parameters showed in all cases the values of Hue° index: 31.55 ± 6.84, 31.09 ± 6.92 and 30.81 ± 6.81.

Statistical analysis showed a positive correlation coefficient in radish root fresh weight comparing the both environments (highland radish and fall radish) (*p*-values < 0.01). Specifically, radish root fresh weights were higher in the first crop with respect to the second crop (values ranged from 49.4 ± 9.8 to 54.3 ± 12.2 g for the first crop and from 28.2 ± 8.6 to 28.3 ± 4.3 g for the second crop). Similar trend was obtained for fresh radish leaves weight, with higher values in the first than second crop (39.3 ± 13.8 to 45.3 ± 11.6 g and 28.6 ± 9.4 to 31.5 ± 7.4 g, with *p*-values < 0.01). For more information, see Fig. S3. The mean moisture of radish in the first crop was 85.2 ± 4.2%, which was greater than that in the second crop (77.3 ± 2.8%), although the difference was not statistically significant (*p*-value > 0.05). These differences found in biomass (mainly fresh weight of roots and leaves) can be attributed to the difference of temperature in both environments (average 21.4 °C and 14.3 °C in the first and second crop, respectively). According to Oh et al. (2022), although the radish roots grow well and without thermal stress with a night/day temperature range between 13 and 19 °C, the optimal temperature range for producing high-quality radish roots of marketable size and weight is 18–24 °C (the same temperature range as the first crop). On the other hand, Kleier et al. (2001) studied the effects of radish root temperature on plant growth. Results indicated a 32% decrease in root weight at 13 °C compared to 18 °C, accompanied by a reduction of leaf area of approximately 0.03 m^2^.

In contrast, no significant difference in the growth of radish roots grown in RE media compared to spiked media (MC and HC) (*p* = 0.40–0.99 for the first and second experiment, respectively) (more information in Fig. S3). A similar trend was obtained for other agronomic parameters such as diameter (*p* = 0.81–0.06), colour (*p*_*IC*_ = 0.08, *p*_*chroma*_ = 0.62 and *p*_*Hue*_ = 0.88) or moisture (*p* = 0.31–0.22). These results suggested that pharmaceutical content in irrigation water does not play a large role in radish quality. Although studies on the impact of pharmaceuticals on crop development and health are limited, the obtained results contrast with those published in the literature. This may be attributed to the fact that in most cases, experiments are conducted with pharmaceutical concentrations higher than those used in this study. As an example, Shenker et al. (2011) reported visible phytotoxic effects in cucumber plants growing under hydroponic culture and exposed to high concentrations of CBZ (> 10 mg/L in irrigation water). These crops showed a reduction in total weight biomass and length (about 50%), as well as the number and size of mature leaves. Xu et al. (2020) reported toxic stress effects on *Cyperus involucratus* plant growth at 1 mg/L of SMX. Türkoğlu et al. (2019) reported that applied ACT concentrations between 50 and 250 mg/kg retarded the root and stem development, increasing the electrolyte leakage and antioxidant enzyme activities in wheat plants. On the other hand, Carter et al. (2015) studied the quality of zucchini leaf plants treated with CBZ. The authors reported a relationship between carbamazepine concentration and visible signs of necrosis. Phytotoxic effects were observed after a week in older leaves (burnt leaf edges and white spots) at concentrations greater than 4 mg of CBZ/kg, accompanied by a decrease in biomass (30–60%).

**Fig. 1:**
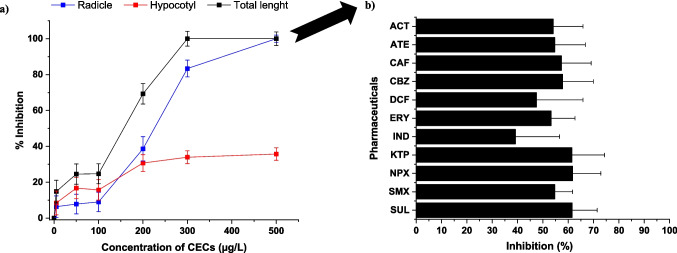
**a** Effects of the mixture of selected pharmaceuticals on the radish seeds’ growth (total, radicle and hypocotyl length). **b** Effects of individual pharmaceuticals on the total length of radish seeds (500 µg/L)

Considering this perspective, in a complementary way, a seed germination toxicity test was performed as a toxicological screening system for assessing the effects of pharmaceuticals in a range of concentrations in radish growth. Specifically, the synergistic effects that can occur when real contaminated wastewater is used for crop irrigation were evaluated (for conditions, see S3). Results suggested a relationship between pharmaceutical concentration and radish growth inhibition. Particularly, a significant deleterious effect on the germination potential of seeds exposed to concentrations higher than 100 µg/L (% inhibition > 25%) was observed, reaching 100% of total length inhibition at 300 µg/L of the mixture of pharmaceuticals (see Fig. 1a). From 100 µg/L, the percentage of radicle inhibition was considerably higher than in hypocotyl, reaching 100% at 500 µg/L. Obtained results are in line with those reported by other authors. Hillis et al. (2011) reported a detailed study on the effects of ten pharmaceuticals on seed germination and root elongation in three plant species, namely, lettuce (*Lactuca sativa*), alfalfa (*Medicago sativa*) and carrot (*Daucus carota*). A decrease in root length and plant growth was observed in almost all cases. On the other hand, Pino et al. (2016) investigated the phytotoxicity of 15 common pharmaceuticals on the germination of lettuce (*Lactuca sativa*) seeds. Twelve of them, including DCF, ACT or SMX, caused inhibitory effects on the radicle (EC_50_ 170–5656 mg/L) and hypocotyl (EC_50_ 188–4558 mg/L) elongation of seeds.

Following the obtained results, and with the aim of evaluating which pharmaceutical could have a greater impact on seeds germination, a second phytotoxicity test was carried out focusing in a concentration of 500 µg/L of each single pharmaceutical. The results in inhibition percentages (Fig. 1b) were classified as follows: (a) 0–25% low toxicity, (b) 25–50% moderate toxicity, (c) 50–75% high toxicity and (d) 75–100% very high toxicity (Bagur-González et al. 2011). Among the 11 pharmaceuticals, two (DCF and IND) showed moderate toxicity (47.49 ± 18.37% and 39.15 ± 17.4%, respectively), while the rest showed high toxicity (from 53.24 ± 9.39 to 61.76 ± 11.12%). Although these percentages suggest a potential phytotoxic effect in seeds, in no case, a percentage of inhibition greater than 75% (very high toxicity) was obtained, suggesting that the mix of pharmaceuticals may have a greater impact on seed germination and thus on the growth of radish crops, with respect to the individual compounds, a similar trend to that reported previously by Yang et al. (2021). According to this study, pharmaceutical mixtures can cause a unique toxicity due to the interactive effect, resulting in stronger synergistic toxicity. On the other hand, Rede et al. (2019) described statistically significant differences in seed germination exposed to a mixture of three pharmaceuticals with respect to the individual exposure of them.

### Impact of irrigation water quality and growth environments on pharmaceutical’s uptake and distribution in plant tissues

**Fig. 2:**
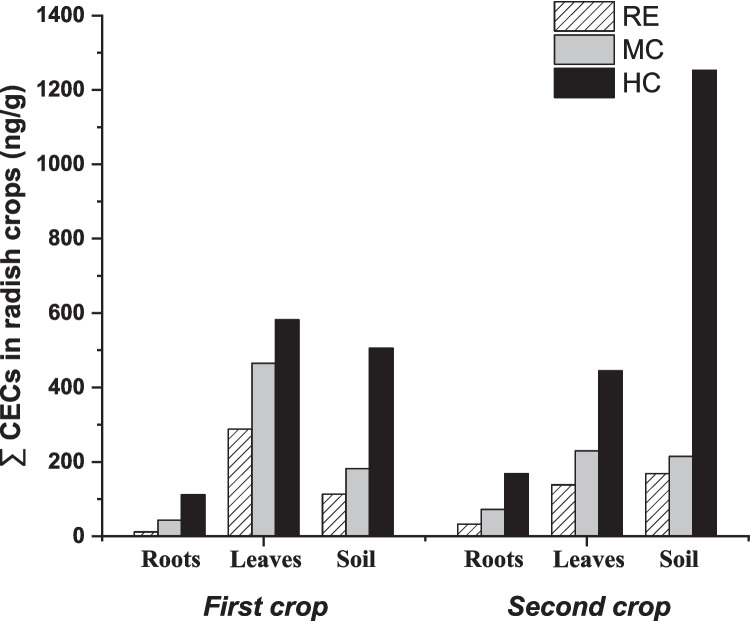
Overall concentrations (ng/g) of pharmaceuticals in radish roots, radish leaves and real soils (0–5 cm of the soil profile)

As shown in Fig. 2, the pharmaceutical concentration (expressed as total sum) in radish tissues as well as in soil is directly related to the quality of irrigation water, suggesting this variable as a key factor in the uptake and accumulation of pharmaceuticals in real crops (soil–plant system). In all cases, the following trend was observed: RE < MC < HC. Specifically, RE concentration ranges were between 11.45 and 32.43 ng/g in roots, between 138.16 and 287.83 in leaves and between 112.94 and 168.23 ng/g in soil. Values of approximately one order of magnitude higher were found for HC (111.63 to 168.54 ng/g in roots, 444.99 to 581.93 ng/g in leaves and 505.53 to 1252.91 ng/g in soil), while intermediate concentration values were found for MC. This trend is in line with that reported by other authors, regardless of crop type, demonstrating the ability of crops to pharmaceuticals uptake. Ponce-Robles et al. (2022) studied the concentration of four pharmaceuticals (from < 0.2 ng/L to 5.1 µg/L) in lettuce crops. Results confirmed a correlation between uptake capacity and pharmaceutical concentration in irrigation water. Benelhadj et al. (2024) studied the uptake of 16 pharmaceuticals in carrot crop. Higher concentrations were obtained when fortified reclaimed water (containing a 100 µg/L of pharmaceuticals) was used for irrigation with respect to non-fortified (control). Carter et al. (2015) studied the uptake capacity of two pharmaceuticals (carbamazepine and verapamil) by zucchini crops, determining that uptake increased in a dose-dependent manner. Similar results were reported by Badar et al. (2022) using spinach as the target crop under three different pharmaceutical concentrations (50 mg/L, 100 mg/L and 200 mg/L).

On the other hand, differences in radish tissues’ pharmaceutical concentration were obtained comparing both environmental grown conditions (first crop, corresponding to highland radish, and second crop, corresponding to fall radish). In the first crop, pharmaceutical concentration in leaves was higher with respect to the second crop, reaching values of 581.93 ng/g when HC reclaimed water was applied, while an inverse trend was obtained for roots (higher concentrations in the second crop). This behaviour can be attributed to the direct relationship between environmental conditions and the evapotranspiration rate of crops (ET) (Pallardy 2007). In general, non-stressed plants grown under the most favourable hot-dry environments (for radish 18–24 °C), as described for the first crop (mean temperature and net radiation of 21.4 °C and 281.6 MJ/m^2^-day, respectively) have a higher water and nutrient uptake capacity with respect to stressed plants and a consequent higher potential for pharmaceutical uptake through roots and transport to aerial tissues, due to their increase in ET (Christou et al. 2019). On the contrary, cold-humid environments such as measured for the second crop (mean temperature and net radiation of 14.3 °C and 143.0 MJ/m^2^-day, respectively) may limit the root–leaf transport due to decreased ET.

About pharmaceutical concentration in agricultural soils, Fig. 2 shows higher values in the second crop compared to the first crop, especially in HC effluents (1252.9 ng/g and 505.5 ng/g, respectively) suggesting that weather conditions may affect soil uptake and/or accumulation capacity. Indeed, several authors have reported that the uptake of pharmaceuticals by crops is largely dependent on their bioavailability/bioaccessibility in soil-pore water near the rhizosphere (Madikizela et al. 2018). In low ET conditions (stressed and/or cold-humid environments), irrigation water can be retained through soil-pore system, resulting in higher accumulation of pharmaceuticals in the soil (mainly in surface layer where radish usually grows) (Qin et al. 2017).

Although the accumulation of pharmaceuticals by the soil–plant system is clear from Fig. 2, it should be noted that only a small percentage of the total pharmaceuticals present in the irrigation water during crop growing was taken up by the radish crop. In this sense, a mass balance was carried out focusing on the most unfavourable conditions regarding the concentration of pharmaceuticals (HC) in irrigation water. Results showed that only 5.3% (first crop) and 3.4% (second crop) of the total pharmaceuticals present in the irrigation water were taken up by radish (specifically 1.0% in roots and 4.3% in leaf for the first crop and 0.9% in roots and 2.5% in leaf for the second crop), while the rest (94.70% and 96.6%, respectively) were lost in the soil system–ambient air, or even metabolized by the plant (see Table S6).

**Fig. 3:**
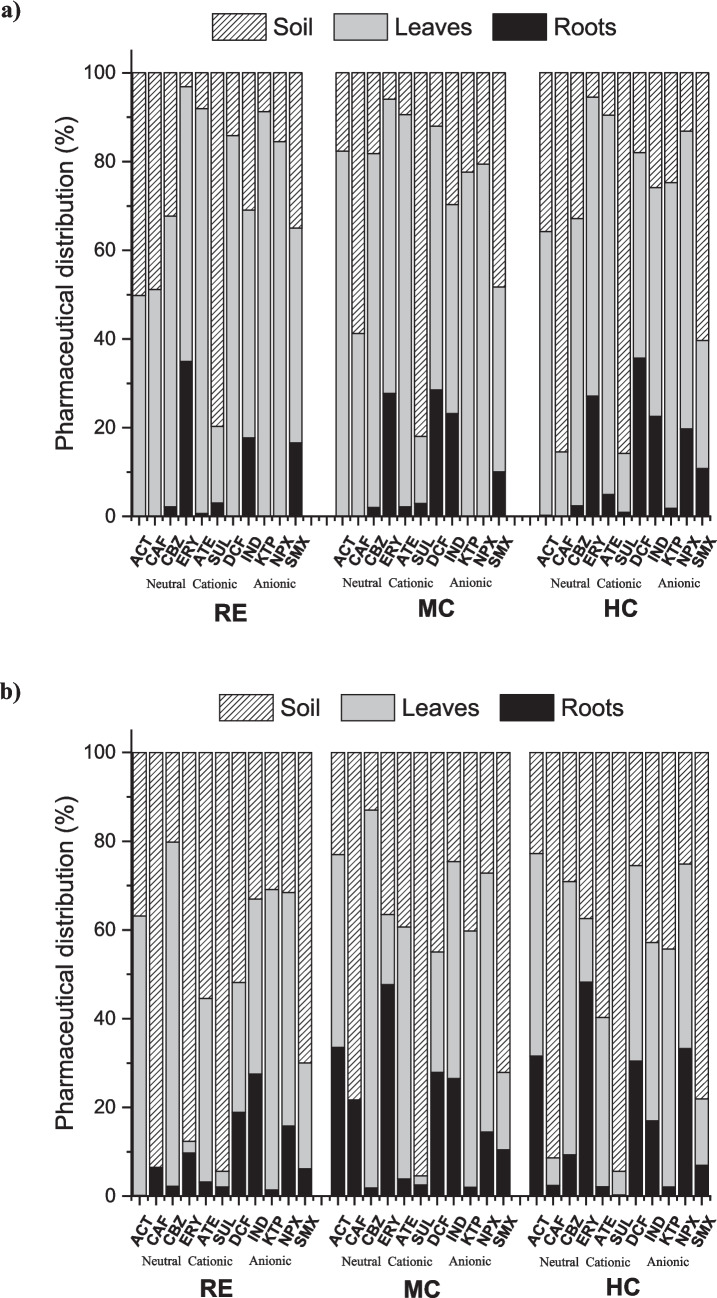
Pharmaceutical distribution in radish tissues: **a** first crop and **b** second crop

Additionally, with the aim to clarify the general distribution trend of each selected pharmaceutical in the soil–plant system under different irrigation water qualities and environmental conditions, the overall distribution (expressed as the total percentage of each individual compound in the soil–plant system (roots + leaves + soil), excluding losses from leaching, degradation and generation of by-products, was studied. Results (Fig. 3) revealed a greater influence of environmental conditions on the total distribution of pharmaceuticals with respect to irrigation water quality, since most compounds had similar behaviour under the three reclaimed waters used. Specifically, the average distribution percentages for the first crop were 9.0 ± 2.4% for roots, 59.2 ± 6.0% for leaves and 31.8 ± 3.8% for soil, while for the second crop, they were 14.2 ± 5%, 35.7 ± 2.3% and 46.1 ± 11.4%. These results suggest, on the one hand, that control of environmental conditions may be a key factor in limiting the pharmaceutical distribution in soil–plant systems and, on the other, that the accumulation of pharmaceuticals in roots (edible part of radish) might be lower than in the rest of the plant tissues, a question of interest for a consumer-level risk analysis. A similar distribution trend (lower concentrations in roots than leaf) had been published by different authors in recent years. Shenker et al. (2011) studied the uptake capacity of CBZ in cucumber plants irrigated with real effluents (concentration of 2.99 µg/L of the selected compound, similar to those applied in this study). The authors reported that most CBZ in the plant tissues (76–84% of total uptake) was detected in the leaves. Hussain et al. (2016) studied the uptake capacity of ciprofloxacin by carrot. Results showed a concentration of 0.10 ± 0.05 ng/g in roots, while higher values were found in leaves (0.48 ± 0.10 ng/g). On the other hand, Pérez et al. (2022) studied the uptake capacity of pharmaceuticals in maize (*Zea mays* plants). After 21 days of exposure time, the concentration of CBZ and atrazine in leaves was higher than in roots (98.4 ± 14.1 ng/g (root) and 251.6 ± 73.9 ng/g (leaf) and 24.8 ng/g ± 7.7 (root) and 4.63 ± 1.3, respectively).

Although these results allow an overview of the performance of the pharmaceuticals in the soil–plant system, it is necessary to highlight that not all compounds presented the same levels of concentration of selected compounds in crops. In general, there are other key factors to be considered in the soil–plant uptake and accumulation, such as the physicochemical properties of each pharmaceutical, which will be discussed in later sections.

### Potential factors influencing the uptake of pharmaceuticals in radish tissues

**Fig. 4:**
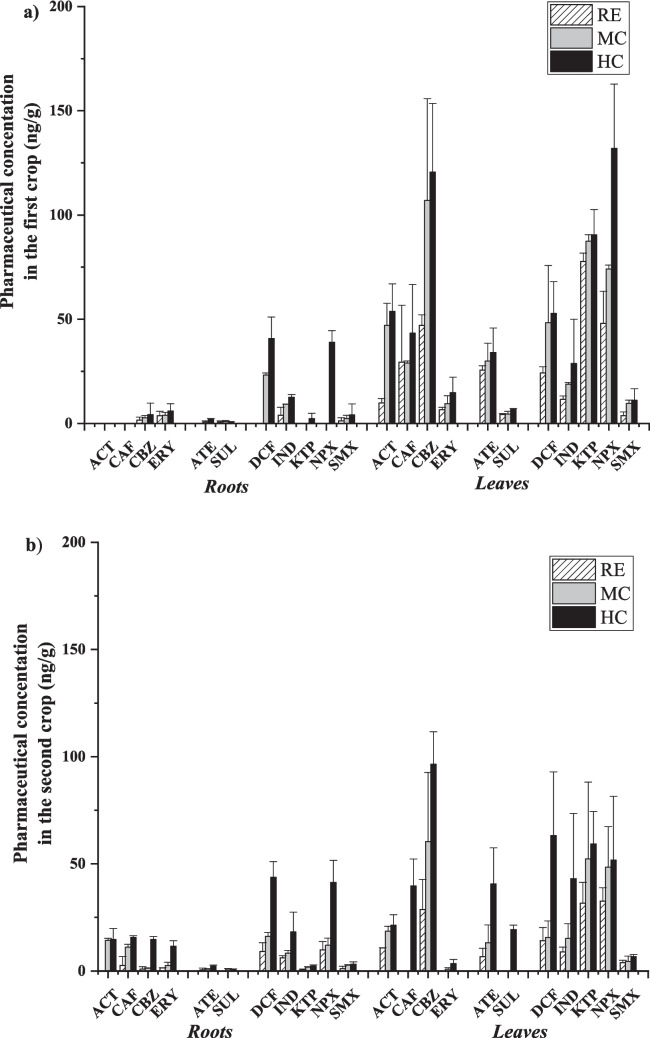
Pharmaceutical concentration in radish tissues (roots and leaves): **a** first crop and **b** second crop

Greater knowledge about the specified concentration levels of each pharmaceutical in crop tissues (including roots and leaves) irrigated under different growing conditions is key to determining potential consequences of reusing wastewater, since each part of the crop could represent a pathway for human exposure to pharmaceuticals (see the “Human health risk from dietary uptake” section). With this in mind, and in addition to the previous section, the average concentration levels of pharmaceuticals under the two experimental growing conditions and the three water qualities are shown in Fig. 4.

All pharmaceuticals were quantified for both radish tissues in the first crop, except ACT and CAF in roots, with lower average concentrations in roots (from MQL to 3.99 ng/g for RE, from MQL to 9.23 ng/g for MC and from 0.47 to 40.75 ng/g) than leaves (from 3.73 to 77.62 ng/g for RE, from 4.54 to 106.95 ng/g for MC and from 6.72 to 120.57 ng/g for HC). For the second crop, all pharmaceuticals were quantified in radish tissues. In this case, as in the first crop, the highest concentrations were found on the leaves. However, these concentrations were slightly lower than the first crop due to the influence of ET, as discussed in the “Impact of irrigation water quality and growth environments on pharmaceutical’s uptake and distribution in plant tissues” section (from MQL to 28.73 ng/g for RE, from MQL to 60.35 ng/g for MC and from 6.67 to 59.32 ng/g in HC).

Regarding the environmental conditions, the compounds that reached the higher concentrations in roots were DCF and NPX (mainly in HC experiments), with average values ranging from 23.23 ± 0.98 to 40.76 ± 10.30 ng/g and < MQL to 38.95 ± 5.58 ng/g, respectively, for the first crop, and from 9.1 ± 4.07 to 43.69 ± 7.36 ng/g and 9.76 ± 3.93 to 41.34 ± 10.3 ng/g, respectively, for the second crop. However, in the leaves, CBZ, NPX, KTP and DCF were the pharmaceuticals that were found at higher concentrations, indicating higher mobility capacity through the plant transpiration stream. On the contrary, ERY, SMX and SUL showed low concentration values in both radish tissues.

In general, plant uptake is thought to be heavily dependent on the physicochemical properties of each pharmaceutical, including water solubility, polarity or octanol–water partition coefficient. Dissociation constants are too important since they can describe whether a chemical is neutral or ionizable at environmentally relevant pH values. Neutral compounds are identified as having a higher capacity for membrane penetration with respect to ionic compounds (Al-Farsi et al. 2017). Therefore, it is expected that these molecules can be taken up and translocated easily via transpiration via the xylem (Dodgen et al. 2015). However, this pattern is not clearly detected for the whole group of neutral compounds under study (ACT, CAF, CBZ, ERY). This can be attributed to their hydrophobicity (log *K*_ow_). It has been reported that, in neutral compounds, the highest uptake by crops is favoured when log *K*_ow_ is between 1 and 4. Compounds with log *K*_ow_ > 4 (very hydrophobic) have limited absorption due to their high affinity for soil, while compounds with log *K*_ow_ < 1 (very hydrophilic) have difficulty moving through the phospholipid membrane of root tissues (Keerthanan et al. 2020). Of the neutral pharmaceuticals under study, only CBZ and ERY have log *K*_ow_ in the appropriate range for uptake. However, lower concentrations were obtained in all cases for ERY with respect to CBZ, due to their high molecular weight, together with its easy mineralization in soils (Shen et al. 2022), factors that may limit its uptake.

For ionic compounds, several authors reported that uptake capacity is closely related to potential attraction or repulsion in the root membrane of crops as well as their ability to interact with soil (Keerthanan et al. 2020). However, in our view, just like neutrals, log *K*_ow_ is a critical factor in radish uptake. As described above, NPX, KTP and DCF were detected at higher concentrations, especially in leaf. These compounds have log *K*_ow_ values of 3.18, 3.12 and 4.51, respectively, close to those reported as optimal for uptake. These compounds, in turn, have a pKa between 4.15 and 4.45. According to Goldstein et al. (2014), these pKa values suggest their partial existence as non-ionic species in the crop rhizoplane, allowing their accumulation in the different plant tissues. Therefore, they could have a similar behaviour to the neutral.

### Root concentration factor and translocation factor

To better understand the accumulation potential of pharmaceuticals in radish tissues under the two environmental grown conditions and the three selected irrigation water qualities (RE, MC and HC), root concentration factor (RCF) and translocation factor (TF) were considered. These factors could be calculated only for those pharmaceuticals that were quantified in the corresponding radish tissues.

**Fig. 5:**
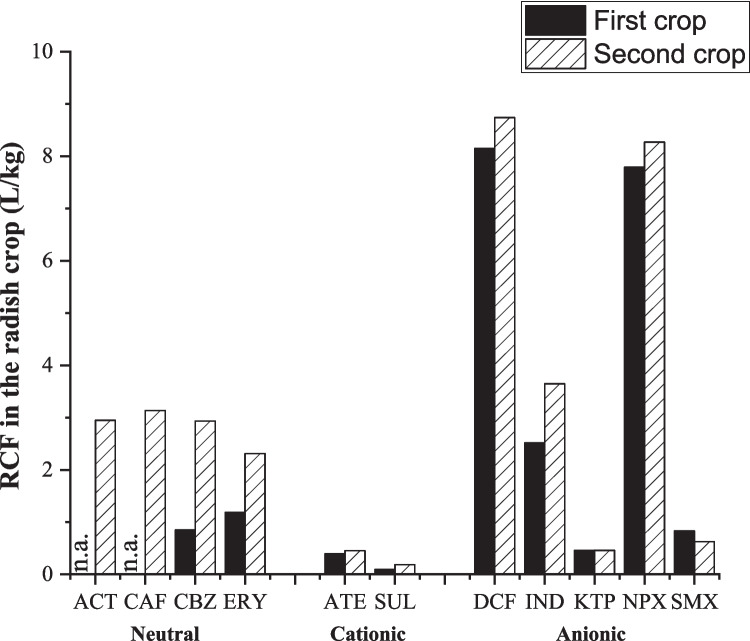
Root concentration factor (RCF) values of pharmaceuticals for higher concentration (HC) in both crops. The values are the average of three samples collected at the irrigation water HC. n.a.: RCF not available because the pharmaceuticals were below the limit of quantification of the method in roots of any crops

The mean RCF in the first crop was lower than in the second crop (see Fig. 5 and Figs. S4–S5), although the difference was not significant due to the wide range of pharmaceuticals under study. Regardless of environmental grown conditions, RCF values in roots irrigated with RE were one order of magnitude higher than those irrigated with MC and two orders higher than those irrigated with HC, demonstrating, according to González García et al. (2018) and Bigott et al. (2020), a clear relationship between the irrigation water quality and the accumulation capacity of pharmaceuticals. Specifically, ranges between 0 and 798.5 L/kg for RE, 0 and 46.5 L/kg for MC and 0 and 8.2 L/kg for HC were obtained in the first crop and between 5.9 and 1952.1 L/kg for RE, 1.7 and 32.3 L/kg for MC and 0.2 and 8.7 L/kg for HC in the second crop.

In general, although the accumulation rates depend on the physicochemical properties of each individual pharmaceutical, RCF values were lower for cationic than for neutral or anionic pharmaceuticals (cationic < neutral < anionic) for the three irrigation water sources. This behaviour is attributed to the great capacity of roots to accumulate anionic compounds compared to other tissues (Wei et al. 2023). In this sense, despite the electrostatic repulsions between anionic compounds and negatively charged cell membranes, these compounds can penetrate the root via water mass flow, resulting in a greater accumulation (Goldstein et al. 2014). Specifically, if we focus on HC (mainly because in RE and MC, there are several compounds below method quantifications limits, so it was not possible to calculate its RCF value) (Fig. 5), highest RCF values were found for DCF and NPX in both growing scenarios, with values of 8.2 L/kg and 7.8 L/kg, respectively, for the first crop and 8.7 L/kg and 8.3 L/kg for the second crop. This could be due to the higher hydrophobic character of both pharmaceuticals with respect to other anionic pharmaceuticals studied, as it has been previously reported that the octanol–water partition coefficient plays an important role in the accumulation and uptake of pharmaceuticals by plant roots (Bigott et al. 2021). In addition, cationic and neutral compounds have a higher ability to move to aerial tissues than anionic, limiting their accumulation in root tissues (de Santiago-Martín et al. 2020). RCF values for cationic and neutral compounds were between 0.1–0.4 and 0.2–1.2 L/kg in the first crop and 0.2–0.5 and 2.3–3.1 L/kg in the second crop, respectively. The same trend was reported previously by several authors. Dodgen et al. (2015) studied the pharmaceutical accumulation in carrot, lettuce and tomato crops, obtaining in all cases higher RCF values for anionic compounds than for cationic compounds. Similarly, Ayala Cabana et al. (2024) reported that anionic compounds such as gemfibrozil, ibuprofen or sulfamethoxazole can accumulate in lettuce roots, while cationic or neutral ones can be found in other crop tissues. Ravichandran and Philip (2021) studied the accumulation of an anionic (DCF), a neutral (CBZ) and a cationic (ATE) compound in two tropical plants. They obtained RCF values of 0.23–0.53 L/kg for diclofenac, 0.14–0.21 L/kg for carbamazepine and 0.07 L/kg for atenolol, indicating that the accumulation in roots of anionic species is significantly higher than that of neutral and cationic species. González García et al. (2018) obtained higher RCF values for DCF than for CBZ in different lettuce varieties irrigated with different concentrations of the two pharmaceuticals.

On the other hand, radishes’ ability to transfer pharmaceuticals from roots to leaves was evaluated using TF, whose values are given in Table 2. All selected pharmaceuticals, except ERY in the second crop, were translocated from roots to leaf tissues (TF values ≥ 1), demonstrating high affinity towards the leaves. This general behaviour may be associated with the high molecular weight of ERY compared to other pharmaceuticals tested (for Mw values, see Table 1). Indeed, large-sized pharmaceuticals (Mw > 400 g/mol) have been shown to have difficulties in translocation due to their exclusion by leaf cell membranes from the crop, resulting in a greater accumulation on roots than on leaves. However, small-sized pharmaceuticals (Mw < 300 g/mol) tend to transport easily to leaves (Chuang et al. 2019). Specifically, it has been reported that pharmaceuticals with small molecule sizes can easily permeate the membrane and, hence, easily come in and go out of the phloem and xylem. On the other hand, compounds with large molecule sizes have low permeability in membranes and therefore cannot be effectively transported in the phloem (Kvesitadze et al. 2015; Wei et al. 2023).

**Table 2:** Translocation factor (TF) values of pharmaceuticals for the three water qualities in both crops

Compound	TF roots-leaves					
	First Crop			Second crop﻿		
	RE	MC	HC	RE	MC	HC
Acetaminophen (ACT)	-	-	-	-	**1.3**	**1.4**
Atenolol (ATE)	-	**41.0**	**17.2**	**13.1**	**14.6**	**18.0**
Caffeine (CAF)	-	-	-	-	-	**2.5**
Carbamazepine (CBZ)	**30.1**	**39.5**	**26.8**	**34.8**	**46.0**	**6.6**
Diclofenac (DCF)	-	**2.1**	**1.3**	**1.6**	**1.0**	**1.4**
Erythromycin (ERY)	**1.8**	**2.4**	**2.5**	-	0.3	0.3
Indomethacin (IND)	**2.9**	**2.0**	**2.3**	**1.4**	**1.8**	**2.4**
Ketoprofen (KTP)	-	-	**39.3**	**49.4**	**28.6**	**25.7**
Naproxen (NPX)	-	-	**3.4**	**3.3**	**4.0**	**1.3**
Sulfamethoxazole (SMX)	**2.9**	**4.1**	**2.7**	**3.8**	**1.7**	**2.1**
Sulpiride (SUL)	**5.7**	**5.1**	**14.2**	-	-	**20.6**

*RE* real effluent; *MC* medium concentration; *HC *higher concentration. The highlighted data are TF values ≥ 1

Higher TF values were obtained for cationic compounds (ATE and SUL) with respect to neutral or anionic, demonstrating the high translocation capacity of this group of pharmaceuticals to the aerial part of crops, a similar trend as reported by Madikizela et al. (2018). According to Goldstein et al. (2014), this behaviour could be attributed to the attraction of pharmaceuticals positively charged with negatively charged cell membranes. Specifically, the authors studied the translocation capacity of different pharmaceuticals in cucumber and tomato. Results demonstrated higher concentrations of compounds positively charged in leaves than roots, resulting in high TF values.

Unexpectedly, CBZ and KTP, despite being one neutral (CBZ) and one anionic (KTP), showed the highest TF values (26.8 to 39.5 for the first crop and from 6.6 to 46.0 for the second crop, and 39.3 for the first crop and 25.7–49.4 for the second crop, respectively). This behaviour could be related to their hydrophobicity. Studies with other organic compounds have shown that the translocation of contaminants is favoured when log *K*_ow_ is between 1 and 4. This can be represented by a Gaussian distribution, where the maximum translocation of chemicals is observed at a log *K*_ow_ of ∼1.78 (Goldstein et al. 2014; Colon and Toor 2016). In this sense, CBZ and KTP have values of *K*_ow_ within this range (2.45 and 3.12, respectively).

Although there are not many detailed studies covering the translocation capacity of the whole group of pharmaceuticals selected in this work, some authors reported similar behaviour of these compounds. Specifically, González García et al. (2019) predicted the accumulation rates of KTP, NPX and ibuprofen (IBU) in lettuce crops. The authors described that the accumulation of these compounds in leaves followed the order KTP > NPX > IBP, while in roots followed the order NPX > IBP > KTP, which results in the most translocated compound being KTP. Similarly, Labad et al. (2021) obtained a higher concentration of ketoprofen in leaves of radish than in roots, where this compound was not detected. On the other hand, higher TF values were reported for CBZ and ATE in radish than in other crops such as lettuce or arugula (Kodešová et al. 2019).

However, it is necessary to bear in mind that although factors such as molecular weight, hydrophobicity or electronic affinity may be key for translocation, this is a complex process, and many other factors must be considered, such as soil type and its specific affinity for pharmaceuticals, the crop variety, the concentration of available organic carbon in soil or the environmental conditions (Zhang et al. 2017a).

### Trend of soil pharmaceutical accumulation and leaching

**Fig. 6:**
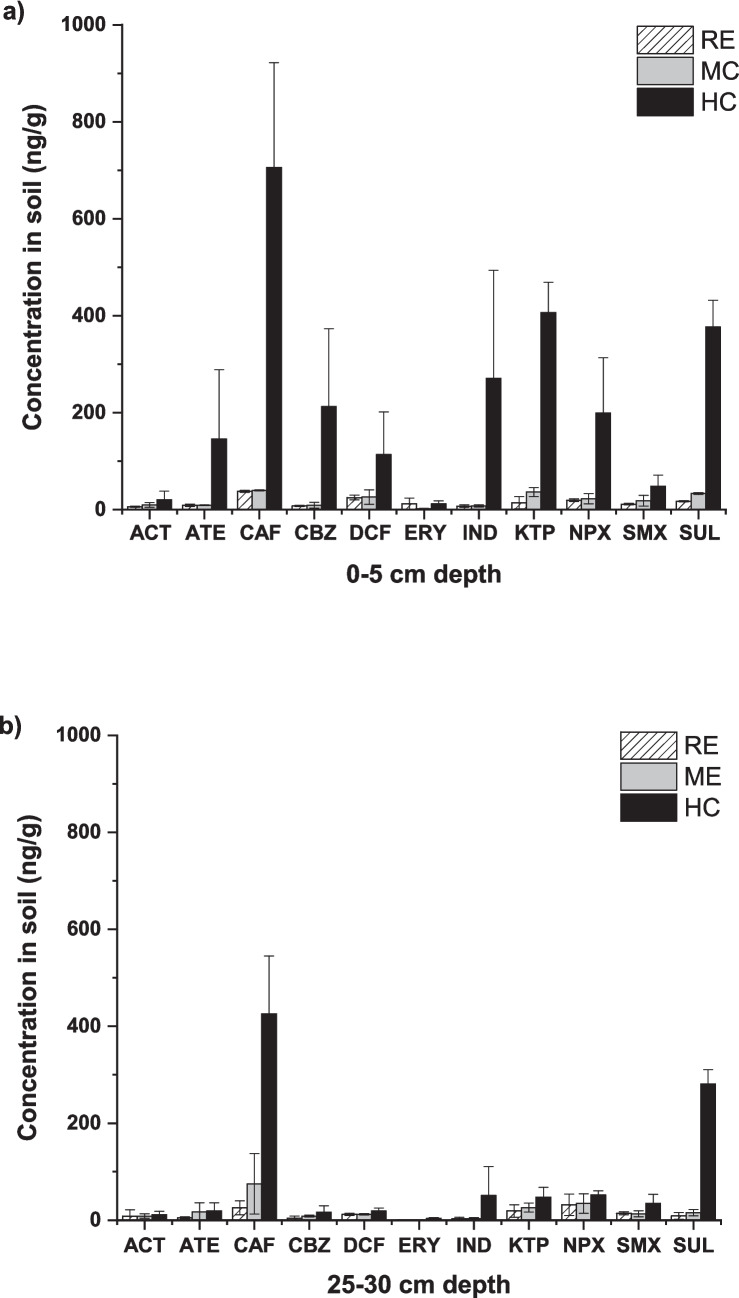
Pharmaceutical concentration in soil collected at the end of the second crop at two depths: **a** 0–5 cm and **b** 25–30 cm

To assess the prolonged exposure to pharmaceuticals in agricultural soils irrigated with reclaimed water of different quality, soil at two depths (0–5 cm and 25–30 cm) collected at the end of the second crop was considered. Data are shown in Fig. 6. In general, CEC concentrations were higher at 0–5 cm (values in the range of 1.9 ± 0.5 and 706 ± 216) than at 25–30 cm depth (whose values ranging from 0.7 ± 0.2 to 426 ± 119), suggesting that the greatest retention of pharmaceuticals occurs in the surface layer where radish normally grows. This could indicate that once pharmaceuticals reach the soil surface, they may be bound together by soil particles, limiting leaching to deeper layers.

As mentioned in the “Impact of irrigation water quality and growth environments on pharmaceutical’s uptake and distribution in plant tissues” section, the concentration values of CECs are related to the quality of the irrigation water; thus, the highest concentration of CECs was obtained when irrigated at a HC. Focusing on this irrigation water quality, the following order was observed for pharmaceutical concentration in real soil samples: CAF > KTP > SUL > IND > CBZ > NPX > ATE > DCF > SMX > ACT > ERY. Among all the compounds studied, CAF showed the highest concentration at both depths, reaching a maximum value of 706 ± 216 ng/g at 0–5 cm depth. This behaviour was expected due to their high sorption coefficient in soils (*K*_oc_ = 741–7762) (Karnjanapiboonwong et al. 2010) and their low concentrations obtained in radish tissues. Bueno et al. (2022) studied the accumulation of a total of 30 CECs in real soil irrigated with reclaimed water containing a mixture of selected CECs, each one at a concentration of 1 µg/L. They found that CAF was one of the pharmaceuticals with the highest accumulation rate in soil. Moreover, high values for the half-life in the environment of between 3.5 and 100 days have been reported for this compound, demonstrating its high persistence (Keerthanan et al. 2021). Williams and McLain (2012) studied the long-term accumulation and leaching of four pharmaceuticals in real soils from different basins at three different depths (between 0 and 50 cm). Caffeine concentration was higher than any of the other pharmaceuticals, independent of the soil clay or organic content.

Indeed, if the *K*_oc_ values reported in the literature (see Table 1) for the pharmaceuticals under study are ordered (CAF > IND > ERY > CBZ > NPX > DCF > ATE > SMX > ACT), a similar trend is observed to that obtained experimentally for all compounds except for ERY, suggesting the *K*_oc_ as a key factor in the accumulation of pharmaceuticals in agricultural soils. According to Shen et al. (2022), this noteworthy behaviour of ERY could be related to its ability to dissipate in soils, as a result of rapid mineralization and transformation into waste. Goulas et al. (2018) studied the behaviour of ERY under different soil exposure scenarios. The authors indicated a 65% mineralization of the compound in long-term exposed soils. On the other hand, Zhou et al. (2023) studied the behaviour of ERY in a soil–lettuce system. A 99.4% of the total ERY applied was degraded in real soils at the lettuce maturity stage.

It should also be noted that, although to our knowledge, no information is available in the literature on *K*_oc_ values for KTP and SUL, these compounds have been found at relatively high concentrations in soil. Therefore, considering the *K*_oc_ as a key factor in pharmaceutical accumulation, it would be expected that its *K*_oc_ values would be above 1400, but below those of the CAF. Despite this assumption, there are other factors that could influence the ability of pharmaceuticals to accumulate in real soils. Zhang et al. (2017b) reported that the high affinity of KTP in soils could be due to the complexation between the carbonyl and carboxyl groups of this pharmaceutical and the metal species present in real soils. Kodešová et al. (2015) indicated that the affinity of pharmaceuticals for soil could be related to their ionic capacity. Li et al. (2013) reported that soil type plays an important role in accumulation rates. Specifically, the authors studied the biodegradability of pharmaceuticals such as CBZ in different soil types, with degradation values ranging from 46 days for sandy clay sludge to 120 days for silty sludge in the case of CBZ. Therefore, further studies would be needed to find out more about the performance of pharmaceuticals in soil and the impact that these compounds could have on agricultural systems.

### Human health risk from dietary uptake

HQ, associated with the intake of target pharmaceuticals through the consumption of the two potentially edible parts of radish (roots and leaves), was covered considering the worst scenario in pharmaceutical uptake (highest concentrations measured in both environmental growing conditions, first and second crop and two age groups (adults, 18–75 years, and infants, 6–11 months)). The acceptable risk threshold was set at a HQ of 1, as described above in the “Health risk assessment” section, where human exposure is equal to the ADI. The greater the magnitude above this threshold, the greater the risk to health.

HQ values were less than 1 in all cases, suggesting negligible exposure risks for individual pharmaceuticals and, therefore, no health risk from ingesting the edible part of radish (for more information, see Table S7). It is worth mentioning that, in general, the EDI value was higher for the leaf than for the root, as a result of the higher concentrations of pharmaceuticals found on the leaf, according to Fig. 2. CBZ was the compound with the highest HQ value, due to its low ADI (340 ng/kg-day) compared to other pharmaceuticals. The highest HQ values for this compound corresponded to radish leaf consumption in infants (0.61 in the first crop and 0.52 in the second crop), due to the low body weight of this group. These results are in line with those reported by other authors. Christou et al. (2017) reported HQ values < 1 when irrigated tomato fruits with tertiary treated wastewater for 3 years, demonstrating that tomato consumption does not pose adverse health effects even in the long term. Sunyer-Caldú et al. (2023) calculated the HQ for adults and children in tomato, carrot and lettuce crops, with HQ values less than 0.016 in all cases. On the other hand, Castaño-Trias et al. (2024) reported that the consumption of lettuce and tomato crops irrigated with reclaimed water under realistic conditions does not pose a human health risk.

Although these results are, a priori, quite favourable and could help to promote confidence between farmers and consumers in the intake of crops irrigated with reclaimed water, it is also necessary to consider the synergistic effects that could be generated by the intake of the pharmaceutical mix through consumption. Under this perspective, the cumulative health hazard index (HI) is estimated using Eq. 5. Estimated values exceed 0.01 but are lower than 0.05 in root consumption by adults, indicating a moderate threat posed by this level of pharmaceuticals to human health, while higher values are obtained for leaf consumption, with values of 0.11 and 0.10 for the first and second crop, respectively. In addition, values higher than 0.05 are obtained for infants in all cases, with values of 0.06–0.14 in roots and 0.89–0.78 in leaf for the first and second experiments, respectively, suggesting a high threat for this age group.

### Environmental risk assessment

**Fig. 7:**
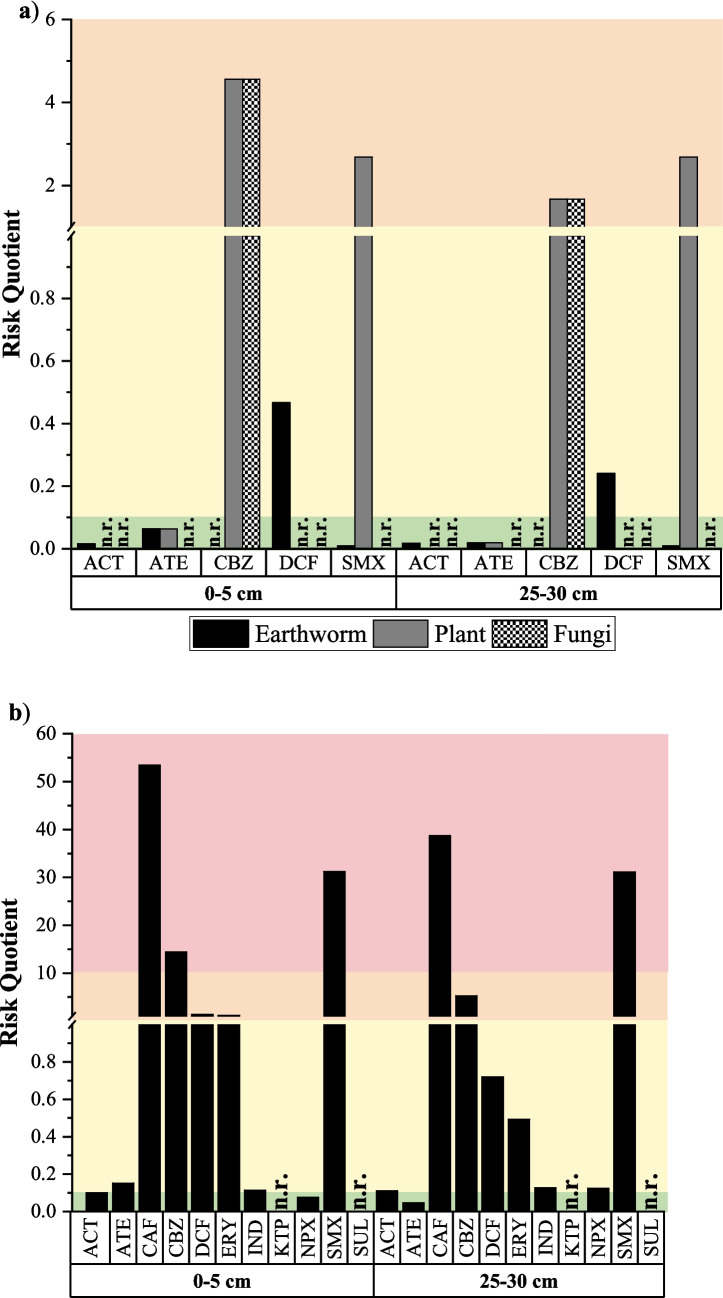
Environmental risk assessment of pharmaceuticals in soil at two different depths depending on the two types of organisms: **a** terrestrial and **b** aquatic

The RQ values for terrestrial and aquatic organisms were calculated at the end of the second crop in both the top (0–5 cm) and bottom (25–30 cm) parts of the soil profile (Fig. 7 and Table S8). As for health risks, it was considered the worst-case scenario of pharmaceutical accumulation (HC). In addition, for terrestrial organisms, only the RQ value could be calculated for five pharmaceuticals because the other compounds were not found in the databases consulted.

In general, RQ values were higher in aquatic organisms, reaching an order of magnitude higher than terrestrial ones. ACT and ATE showed no significant risk for any terrestrial and aquatic organism studied (although ACT values for plants and fungi and ATE for fungi were not available), with RQ values lower than 0.1 in all cases, suggesting no adverse effects on biodiversity. The low toxicity of these compounds in the aquatic environment is supported by other studies, reporting RQ values of ACT and ATE less than 0.1 (Gros et al. 2010; Afonso-Olivares et al. 2017; Singh et al. 2024).

CBZ showed the highest RQ in terrestrial organisms (data not provided for earthworms), with values for plants and fungi of 4.56 at 0–5 cm and 1.67 at 25–30 cm for both organisms, indicating a moderate risk. However, for aquatic organisms, CBZ showed a high risk at 0–5 cm (RQ = 14.4), while moderate risk was calculated at 25–30 cm (RQ = 5.28). RQ values > 1 of this pharmaceutical for invertebrate’s acute test on limed sludge were reported by Camotti Bastos et al. (2020), who suggested an environmental risk from direct contact between organic waste containing this compound and organisms such as algae, fish and plants, as a consequence of their transfer from soil to water by leaching.

DCF showed a low risk for the only terrestrial organism reported (earthworm), with RQ value of 0.47 in the top layer and 0.24 in the bottom, while for aquatic organisms, it exhibited a moderate risk in the upper soil (RQ = 1.39) and a low risk in the lower soil (RQ = 0.72). Other studies reported significant risks of this compound to the aquatic ecosystems, being more toxic to fish species than to algae or invertebrates (Garrido et al. 2016; Papageorgiou et al. 2016).

SMX showed a negligible risk to earthworms (RQ = 0.01 in both layers) and moderate risk to plants (RQ = 2.69 in top layer and RQ = 2.68 in bottom layer), with no data reported on fungi. In contrast, for aquatic organisms, SMX showed high risk with an RQ value of 31.2 in both layers. High RQ values of this antibiotic have been reported in the literature. Chen et al. (2021) studied the ecological risk of a total of 26 PPCPs, including SMX, in two different times of year. They found the highest risk for aquatic organisms with this compound, with an RQ of 579 in summer and 252 in autumn. Rivera-Jaimes et al. (2018) calculated RQ values for a series of pharmaceuticals in surface water and effluent wastewater, obtaining RQ values of SMX of 45.0 and 74.4, respectively.

Given the high concentration of CAF detected in soil and the absence of PNEC_SOIL_ values for terrestrial organisms, it is of interest to consider its risk in aquatic organisms. Among all reported compounds, CAF showed the highest risk quotient in both the top and bottom layers, with RQ values of 53.5 and 38.7, respectively. Other authors have reported RQ values higher than 1 for algae (Thomaidi et al. 2015), crustaceans (Arsand et al. 2023) and fish (Manjarrés-López et al. 2024), which demonstrates that it is a compound that can lead to significant environmental impact.

## Future remarks, directions and approaches for managing and reducing risks

Although the results obtained in the study have allowed us to understand the performance of the selected pharmaceuticals on real radish crops, as well as the potential effects on health and the environment, many challenges still remain. In particular, the study covers a wide range of pharmaceuticals from various therapeutic groups. However, there are thousands of organic contaminants that can accumulate in crop tissues and soils. This also includes compounds that have not yet been identified and may be present in the environment as well as in treatment plants, which are currently unknown. This may include by-products that can be generated from degradation, or interaction with the soil plant system. This has implications for the continuous need for validation of analytical protocols and the use of non-objective strategies in high-resolution analytical equipment.

Another essential point to be considered is that the quality of reclaimed water for irrigation may be different according to the treatment applied in the WWTP. Under our perspective, the best way to minimize the presence of pharmaceuticals in irrigation water (and thus their uptake into crops as well as their accumulation in soil) would be the installation of advanced quaternary treatments specifically design for pharmaceutical removal. In recent years, a lot of effort has gone into developing advanced technologies. However, the difficult to scale small prototypes into large installations, jointly other factors such as high prices of installation and maintenance, make this task difficult. Economic and political incentives could be part of the solution to drive the implementation of such systems.

From an agronomic point of view, and although the reuse practices are a reality, there are doubts on the part of farmers and consumers in the management and consumption of products irrigated with these effluents. Practical guidelines for the management of regenerated water, including measures to limit the presence of pharmaceuticals are necessary in the short term. These guidelines could include screening strategies (to check which pharmaceuticals would be likely to reach the soil-plant system), protection actions for farmers and consumers (to avoid direct contact with regenerated water), crop/variety irrigation restrictions, the adoption of a more efficient irrigation systems (such as deficient irrigation), etc.

The main questions to be considered are: What compounds can reach my crop? What is the probability of being absorbed by the soil plant system? If so, at what level? What are the associated risks?

Although the field of research in terms of quantifying and understanding the health and environmental impacts of the use of reclaimed water in agriculture is still quite wide, studies such as that developed in this work are key to the creation of databases and the establishment of priority chemical lists. This may lead in the short-long term to the create and/or improve practical guidelines and regulations for the reuse of reclaimed water in agriculture.

## Conclusion

Irrigation with regenerated water from EDAR requires specific attention when considering the risk to both health and the environment that may result from the presence of pharmaceuticals in real crops. In this work, the uptake, accumulation and translocation capacity of 11 pharmaceuticals commonly found in reclaimed water was evaluated by monitoring them in real radish crops growing under two environmental conditions.

Results suggested that pharmaceuticals do not play a large role in radish quality at concentration in irrigation water lower than 100 µg/L. However, a direct relationship between pharmaceutical concentration in the soil-plant system and the irrigation water quality and ET was obtained, suggesting that pharmaceutical concentration in reclaimed water and environmental conditions may be key to uptake.

Regardless of the growing environment conditions, leaves showed a higher pharmaceutical concentration than roots, due to translocation of contaminants from root to leaf. Log Kow played a significative role in pharmaceutical uptake and translocation (when log Kow was between 1 and 4).

In general, Koc can be useful for predicting accumulation of pharmaceuticals in soils irrigated with regenerated water, except for compounds susceptible to very rapid mineralization such as ERY.

Environmental risks were calculated to give a clear picture of the impact of reclaimed water in real agricultural soils. Overall, RQ values were an order of magnitude higher in aquatic than terrestrial organisms. It was found that CAF, CBZ and SMX are the compounds that could have a higher impact on aquatic organisms, while CBZ and SMX could have a higher impact on terrestrial organisms.

The assessment of health risks, considering the worst possible exposure scenario for each pharmaceutical through consumption of radish, did not involve any risk to humans. However, more studies focusing on the synergistic effect between pharmaceuticals are needed for a real assessment of potential risks to human health and the environment.

In general, obtained results showed that, despite the urgent need for a transition to more sustainable agricultural production/consumption and food systems through the use of reclaimed water, knowledge of irrigation water quality and appropriate environmental and health risk studies could be essential to ensure safe reuse.

## Supplementary Information

Below is the link to the electronic supplementary material.

MOESM 1 (DOCX 450 KB)

## Data Availability

This is not applicable.

## Glossary

ACT: Acetaminophen
ADI: Acceptable diary intake
AF: Assessment Factor
ATE: Atenolol
CAF: Caffeine
CBZ: Carbamazepine
CECs: Contaminants of Emerging Concern
d.w.: Dry weight
DCF: Diclofenac
EDI: Estimated diary intake
ERY: Erythromycin
ET: Evapotranspiration rate
f.w.: fresh weight
HC: Higher Concentration
HQ: Hazard Quotient
IND: Indomethacin
K_oc_: Organic carbon to water partition coefficient
K_ow_: Octanol–water partition coefficient
KTP: Ketoprofen
L(E)C_50_: Short-term toxicity test
MC: Medium Concentration
MEC_SOIL_: Maximum Environmental Concentration in soil
MQL: Method Quantification Limit
NPX: Naproxen
NSAIDs: Non-Steroidal Anti-Inflammatory Drugs
pK_a_: Acid dissociation constant
PNEC_SOIL_: Predicted No-Effect Concentration in soil
PNEC_WATER_: Predicted No-Effect Concentration in aquatic environment
RCF: Root Concentration Factor
RE: Real Effluent
RH: Relative humidity
RQ: Risk Quotient
R_n_: Net radiation
SMX: Sulfamethoxazole
SPAC: Soil-plant atmosphere continuum
SUL: Sulpiride
Tª: Air temperature
TF: Translocation Factor
QuEChERS: Quick Easy Cheap Effective Rugged and Safe extraction
WWTPs: Wastewater Treatment Plants
