# Supplementary material for: Environmental and health impacts of pharmaceuticals in radish crops irrigated with reclaimed water
Source: Environ Sci Pollut Res Int. 2025 Aug 27;32(35):20920–43. doi: 10.1007/s11356-025-36864-9 (PMC12443892; doi:10.1007/s11356-025-36864-9)
Supplement: Supplementary file 1 — (DOCX 450 KB) [file 11356_2025_36864_MOESM1_ESM.docx]

**SUPPLEMENTARY INFORMATION**

**GENERAL INFORMATION**

**S1. HR-QTOF-MS analysis**

Samples were analysed using an Acquity UPLC I-Class System coupling with an HR-QTOF-MS maXis Series (Bruker Daltonik GmbH, Germany), equipped with an ACQUITY BEH C18 (100 mm × 2.1 mm, 1.7 μm) analytical column. A liquid chromatography-quadrupole time-of-flight mass spectrometry (UPLC-QqTOF-MS) system was used in order to identify and quantify the pharmaceutical compounds selected in this study. Specifically, LC separation was achieved using an ACQUITY I-Class UPLC system (Waters Corporation, Milford, MA, USA) coupled with an ACQUITY BEH C18 (100 mm x 2.1 mm, 1.7 µm) column. Mobile phases were 0.1% formic acid in MilliQ water (solvent A) and 0.1 % MeOH (solvent B). The gradient elution programme used from 5% to 95% of solvent B: Initially was increased from 5% to 25% in 1.50 min, after increasing from 25% to 80%, kept at 80% during 1 min, increased in 8 min and from 80% to 95% in 1 min and finally returned to its initial conditions in 4 min. The total analysis run time was 15.50 min. The flow rate was adjusted at 0.25 mL/min and 10 µL of sample was injected. The LC system was connected to a Bruker Daltonics, maXis q-TOF mass spectrometer equipped with an electrospray ion source (Bruker Daltonics, Bremen, Germany). The ion source settings were as follows: nitrogen was used as nebulizer, drying and collision gas; the nebulizer pressure was 2 bars, the drying gas flow was 8 L/min, the dry gas temperature was 200 ºC and the capillary voltage was 4500 V. The system worked via TOF MS survey scan (resolving power ≥ 55000 FWHM). The target pharmaceuticals were identified and reported from accurate-mass scan data using the software Target Analysis (1.3) and Data Analysis (4.2) from Bruker.

**S2. Linearity, MQL and recovery determination**

Quantitation of target pharmaceuticals was achieved applying external matrix-matched calibration curves, using solutions of selected compound in methanol at five different concentrations, ranging from 0.01 µg/L to 100 µg/L. Linearity was considered as acceptable when the determination coefficients (*R*^2^) were ≥ 0.9.

For the Method quantification limits (MQL) calculation, the concentration of pharmaceuticals in each matrix (roots, leaves and soil) that give a signal to noise ratio of 10 was considered. The concentrations used were the 3 lowest points of matrix-matched calibration curves and these values were corrected by dilution factors and recovery percentages to achieve MQL values.

Percent recovery values were determined by comparing spiked samples before and after QuEChERS extraction (n=3) by adding 100 μg/L of each pharmaceutical. Satisfactory mean recovery values were considered for almost all compounds in the 70-120% range with associated precision RSD ≤ 20%.

**S3. Seed germination toxicity test**

The bioassays involved the exposure of radish seeds to different concentrations of selected pharmaceuticals. For the experiments, 10 commercially available radish seeds were transferred to a Petri dish (10 mm diameter) containing a Whatman filter and 5 mL of real wastewater containing the selected pharmaceuticals. Petri dishes were sealed and placed in a germination chamber at 20 ± 1ºC. The exposure time was five days (120 h).

The root lengths of germinated seeds were evaluated considering three different root sections: the hypocotyl, the radicle and total length (hypocotyl + radicle), to verify the most sensitive metric to assess toxicity (Campagna-Fernandes et al., 2016).

Two different tests were conducted. In the first of these, the percentage of inhibition of seed growth was evaluated at six different concentrations of the mixture of pharmaceuticals (concentration of pharmaceuticals in real Effluents, 50 µg/L, 100 µg/L, 200 µg/L, 300 µg/L, and 500 µg/L), which allowed an assessment of the synergistic effects that can occur when contaminated real water is used for crop irrigation. In the second trial, the percentage of seed growth inhibition was evaluated at a concentration of 500 in contact with each of the selected pharmaceuticals.

In the second trial, seed growth in contact with a solution containing 500 µg/L of each pharmaceutical was evaluated. This allowed the impact on growth to be assessed independently for each of the pharmaceutical.

Experiments were performed in triplicate for each condition (a total of 51 tests). The experiments included negative controls (with distilled water) and positive controls (with a nutrient solution).

**FIGURES**

**Fig S1.** Distribution of the radish crops on the plot.


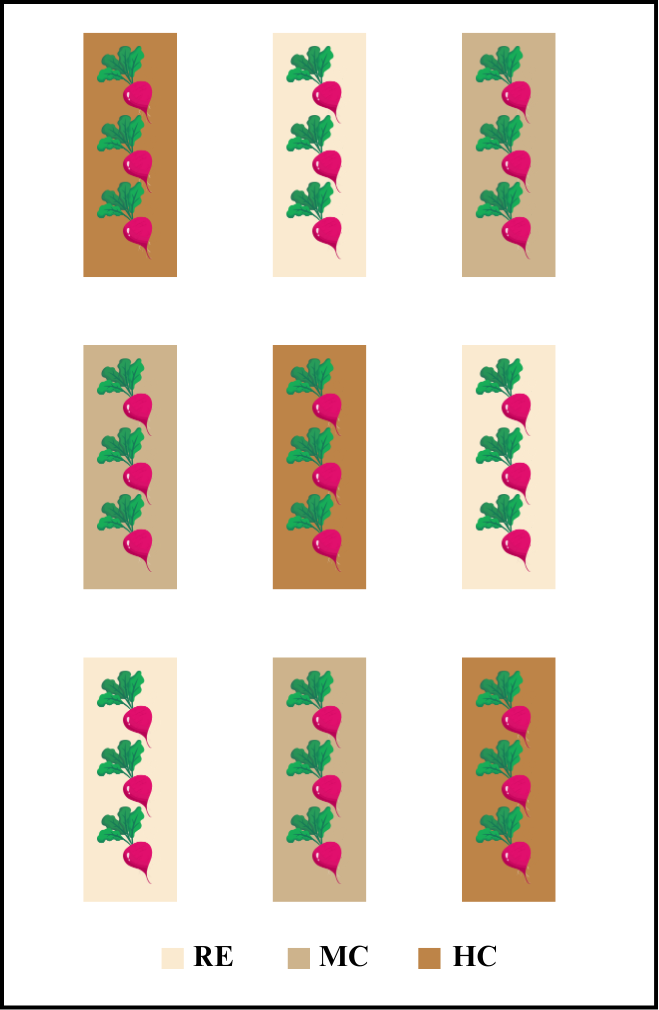


**Fig S2.** QuEChERS extraction methods for pharmaceutical analysis in the matrices studied (radish roots, leaves and soils).


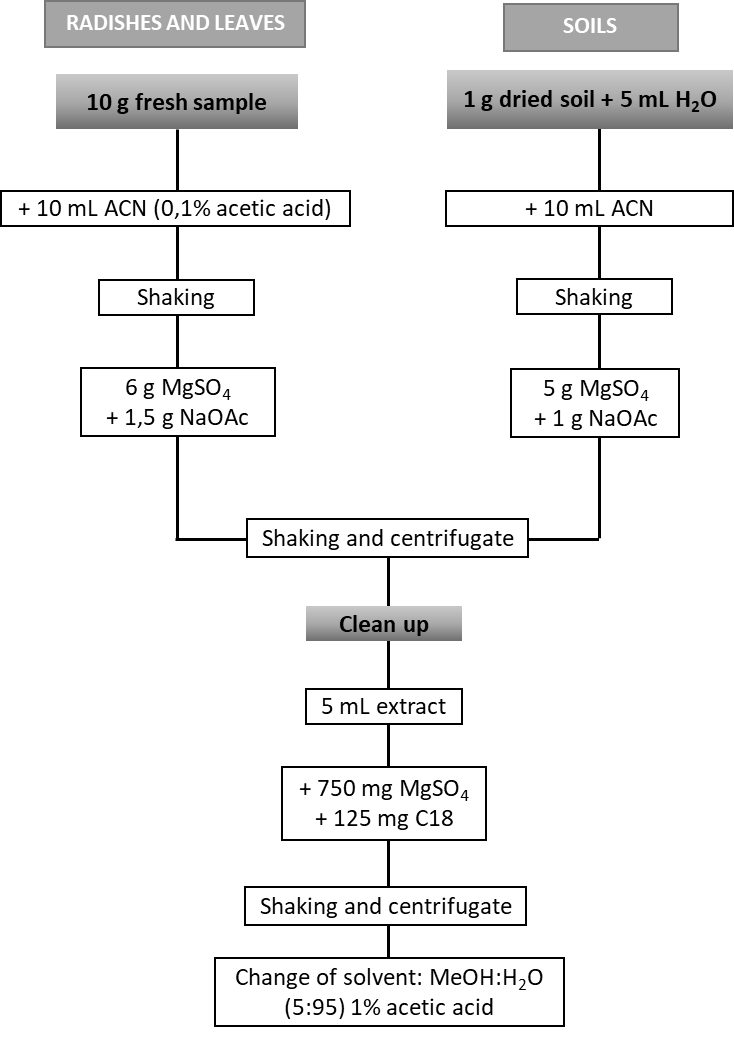


**Fig. S3**. Radish quality parameters under different environmental conditions (highland and fall radish, corresponding to first and second crop, respectively: a) radish roots fresh weight; b) Moisture of fresh roots; c) radish leaves fresh weight.

**Fig. S4.** Root concentration factor (RCF) values of pharmaceuticals for real effluent (RE) in both crops. The values are the average of three samples collected at the irrigation water RE.

**Fig. S5.** Root concentration factor (RCF) values of pharmaceuticals for medium concentration (MC) in both crops. The values are the average of three samples collected at the irrigation water MC.

**TABLES**

**Table S1.** Physicochemical characteristics of WWTP effluents during the experimental period (n = 20).

| **Parameter** | **Value** |  | **Parameter** | **Value** |
| --- | --- | --- | --- | --- |
| ***pH*** | 7.43 ± 0.15 |  | ***F^-^ (mg/L)*** | 0.18 ± 0.03 |
| ***Turbidity*** | 2.66 ± 1.60 |  | ***Cl^-^ (mg/L)*** | 243.57 ± 47.13 |
| ***Electrical Conductivity (EC, dS/m)*** | 1.57 ± 0.15 |  | ***NO_2_^-^ (mg/L)*** | 1.38 ± 0.08 |
| ***DBO_5_ (mg/L)*** | 3.25 ± 2.03 |  | ***Br^-^ (mg/L)*** | 0.38 ± 0.08 |
| ***Total Nitrogen (mg/L)*** | 9.42 ± 1.82 |  | ***NO_3_^-^ (mg/L)*** | 9.93 ± 5.74 |
| ***Dissolved organic carbon (mg/L)*** | 11.67 ± 1.39 |  | ***PO_4_^3-^ (mg/L)*** | 9.08 ± 2.91 |
| ***Total Solids (mg/L)*** | 5.50 ± 2.00 |  | ***SO_4_^2-^ (mg/L)*** | 200.82 ± 30.91 |
| ***Transmittance (%)*** | 53.69 ± 4.58 |  | ***SAR^*^*** | 4.90 ± 1.01 |
|  |  |  | ***E. Coli (CFU/mL)*** | <10 |

**SAR: Sodium adsorption ratio*

**Table S2.** Pharmaceutical content and detection factor in WWTP effluents (RE) during the experimental period (n = 8).

| **Compound** | **MQL* (μg/L)** | **Concentration: Min. and Max. values**  **(μg/L)** | **Frequency of Detection (FoD)** |
| --- | --- | --- | --- |
| ***ACT*** | 0.01 | <MQL-0.09 | 8/8 (100%) |
| ***ATE*** | 0.21 | <MQL-0.21 | 8/8 (100%) |
| ***CAF*** | 0.03 | <MQL-0.12 | 8/8 (100%) |
| ***CBZ*** | 0.01 | 0.01-0.25 | 8/8 (100%) |
| ***DCF*** | 0.01 | 0.05-0.82 | 8/8 (100%) |
| ***ERY*** | 0.01 | <MQL-0.05 | 8/8 (100%) |
| ***IND*** | 0.01 | 0.01-0.13 | 8/8 (100%) |
| ***KTP*** | 0.01 | <MQL-0.89 | 8/8 (100%) |
| ***NPX*** | 0.01 | 0.08-1.47 | 8/8 (100%) |
| ***SMX*** | 0.04 | <MQL-0.05 | 8/8 (100%) |
| ***SUL*** | 0.01 | <MQL-0.27 | 8/8 (100%) |

**MQL: Method quantification limit*

**Table S3.** Physical-chemical soil properties.

| **Parameter** | **Value** |
| --- | --- |
| **Texture** | **Sandy-Loamy** |
| **Sand (%)** | **4.2** |
| **Silt (%)** | **64.8** |
| **Clay (%)** | **30.9** |
| **Total Organic matter (g/100g)** | **2.3** |
| **C/N ratio** | **11.5** |
| **pH** | **7.9** |
| **Ca (g/100 g)** | **18.6** |
| **K (g/100 g)** | **0.9** |
| **Mg (g/100 g)** | **1.3** |
| **Na (g/ 100 g)** | **0.3** |
| **Fe (mg/kg)** | **16962** |
| **Mn (mg/kg)** | **311.3** |
| **B (mg/kg)** | **39.5** |
| **Cu (mg/kg)** | **20.3** |
| **Zn (mg/kg)** | **45.3** |

**Table S4.** Agricultural and environmental conditions for radishes under the two growth scenarios (First and Second crop).

|  |  | **First crop** | **Second crop** |
| --- | --- | --- | --- |
| **Crop conditions** | **Plant density (plants/m^2^)** | 8 | 8 |
|  | **Growing days** | 21 | 30 |
|  | **Total irrigation wastewater (m^3^)** | 2.87 | 2.77 |
| **Greenhouse conditions** | **Tª, _mean_ (ºC)** | 21.4 | 14.3 |
|  | **RH, _mean_ (%)** | 63.4 | 67.4 |
|  | **R_n, mean_ (MJ/m^2^-day)** | 281.57 | 143.04 |

***** *RH: Relative Humidity; R_n_: Net Radiation*

**Table S5.** Linear range, recovery and method quantification limits (MQLs) of selected pharmaceuticals in the three selected matrices (radish roots, radish leaves and soil).

| **Compound** | **Linearity** | | | **MQL* (ng/g)** | | | **Recovery (% ± SD)** | | |
| --- | --- | --- | --- | --- | --- | --- | --- | --- | --- |
|  | **Roots** | **Leaf** | **Soil** | **Roots** | **Leaf** | **Soil** | **Roots** | **Leaf** | **Soil** |
| ***ACT*** | 0.998 | 0.992 | 0.995 | 0.2 | 1.4 | 17.0 | 79 ± 12 | 89 ± 9 | 88 ± 8 |
| ***ATE*** | 0.994 | 0.994 | 0.997 | 0.4 | 1.4 | 3.0 | 78 ± 3 | 84 ± 2 | 79 ± 4 |
| ***CAF*** | 0.999 | 0.992 | 0.992 | 2.0 | 3.0 | 25.0 | 113 ± 7 | 95 ± 8 | 73 ± 13 |
| ***CBZ*** | 0.998 | 0.929 | 0.999 | 0.3 | 0.3 | 5.0 | 91 ± 2 | 98 ± 6 | 105 ± 9 |
| ***DCF*** | 0.960 | 0.993 | 0.996 | 0.3 | 0.1 | 4.0 | 88 ± 5 | 94 ± 7 | 77 ± 8 |
| ***ERY*** | 0.999 | 0.990 | 0.997 | 0.2 | 0.5 | 1.0 | 118 ± 2 | 92 ± 2 | 83 ± 2 |
| ***IND*** | 0.999 | 0.994 | 0.998 | 0.4 | 1.0 | 3.0 | 84 ± 2 | 75 ± 5 | 70 ± 10 |
| ***KTP*** | 0.998 | 0.994 | 0.997 | 0.4 | 0.2 | 4.0 | 108 ± 11 | 100 ± 6 | 93 ± 10 |
| ***NPX*** | 0.994 | 0.998 | 0.990 | 0.3 | 0.3 | 20.0 | 114 ± 6 | 85 ± 9 | 72 ± 2 |
| ***SMX*** | 0.998 | 0.992 | 0.993 | 0.6 | 1.1 | 1.0 | 117 ± 3 | 101 ± 3 | 81 ± 2 |
| ***SUL*** | 0.998 | 0.992 | 0.999 | 0.5 | 1.3 | 1.0 | 102 ± 1 | 98 ± 1 | 75 ± 1 |

**MQL: Method quantification limit*

Table S6. Mass balance of pharmaceuticals in selected crop growing experiments (First and Second crop) under high concentration (HC) conditions.

|  | Total accumulation in radish tissues (%) | | Loses (%) |
| --- | --- | --- | --- |
|  | Radish roots | Radish leaves | Accumulation in soil system/ambient air/others |
| First Crop | 1.03 | 4.28 | 94.70 |
| Second Crop | 0.89 | 2.52 | 96.58 |

**Table S7.** Human health risk assessment of pharmaceuticals in adults and infants ingesting roots and leaves of the first and second crop.

| **Contaminant** | **Adults (18-75 years)** | | | | | | | | | **Infants (6-11 months)** | | | | | | | | | | **ADI**  **(ng/kg-day)** | **Literature** |
| --- | --- | --- | --- | --- | --- | --- | --- | --- | --- | --- | --- | --- | --- | --- | --- | --- | --- | --- | --- | --- | --- |
|  | **First crop** | | | | **Second crop** | | | | | **First crop** | | | | | **Second crop** | | | | |  |  |
|  | **Root** | | **Leaf** | | **Root** | | | **Leaf** | | **Root** | | | **Leaf** | | **Root** | | | **Leaf** | |  |  |
|  | **EDI (ng/kg-day)** | **HQ** | **EDI (ng/kg-day)** | **HQ** | **EDI (ng/kg-day)** | **HQ** | **EDI (ng/kg-day)** | | **HQ** | **EDI (ng/kg-day)** | **HQ** | **EDI (ng/kg-day)** | | **HQ** | **EDI (ng/kg-day)** | **HQ** | **EDI (ng/kg-day)** | | **HQ** |  |  |
| ***ACT*** | 0.05 | <0.01 | 12.14 | <0.01 | 3.32 | <0.01 | 4.81 | | <0.01 | 0.40 | <0.01 | 98.14 | | <0.01 | 26.87 | <0.01 | 38.91 | | <0.01 | 5.0E+04 | Patel et al., 2019 |
| ***ATE*** | 0.45 | <0.01 | 7.68 | <0.01 | 0.51 | <0.01 | 9.18 | | <0.01 | 3.61 | <0.01 | 62.07 | | 0.03 | 4.13 | <0.01 | 74.19 | | 0.04 | 2000 | Bruce et al., 2010 |
| ***CAF*** | 0.00 | <0.01 | 9.78 | <0.01 | 3.54 | <0.01 | 8.97 | | <0.01 | 0.00 | <0.01 | 79.03 | | <0.01 | 28.60 | <0.01 | 72.50 | | <0.01 | 2.5E+06 | Nawrot et al., 2003 |
| ***CBZ*** | 0.96 | <0.01 | 25.74 | <0.01 | 3.31 | 0.01 | 21.77 | | 0.06 | 7.75 | 0.02 | 208.13 | | 0.61 | 26.77 | 0.08 | 176.01 | | 0.52 | 340 | Bruce et al., 2010 |
| ***DCF*** | 9.20 | <0.01 | 11.92 | <0.01 | 9.86 | <0.01 | 14.26 | | <0.01 | 74.35 | <0.01 | 96.36 | | <0.01 | 79.70 | <0.01 | 115.28 | | <0.01 | 6.7E+04 | Bruce et al., 2010 |
| ***ERY*** | 1.34 | <0.01 | 3.33 | <0.01 | 2.61 | <0.01 | 0.77 | | <0.01 | 10.84 | <0.01 | 26.95 | | 0.01 | 21.07 | <0.01 | 6.25 | | <0.01 | 5.0E+03 | Patel et al., 2019 |
| ***IND*** | 2.84 | <0.01 | 6.50 | <0.01 | 4.12 | 0.01 | 9.72 | | 0.01 | 22.98 | 0.03 | 52.52 | | 0.07 | 33.27 | 0.05 | 78.62 | | 0.11 | 710 | Patel et al., 2019 |
| ***KTP*** | 0.52 | <0.01 | 20.42 | 0.02 | 0.52 | <0.01 | 13.38 | | 0.01 | 4.20 | <0.01 | 165.09 | | 0.17 | 4.21 | <0.01 | 108.21 | | 0.11 | 1000 | Patel et al., 2019 |
| ***NPX*** | 8.79 | <0.01 | 29.77 | <0.01 | 9.33 | <0.01 | 11.68 | | <0.01 | 71.05 | <0.01 | 240.72 | | <0.01 | 75.41 | <0.01 | 94.43 | | <0.01 | 5.7E+05 | Bruce et al., 2010 |
| ***SMX*** | 0.94 | <0.01 | 2.50 | <0.01 | 0.71 | <0.01 | 1.50 | | <0.01 | 7.58 | <0.01 | 20.25 | | <0.01 | 5.70 | <0.01 | 12.16 | | <0.01 | 5.1E+05 | Bruce et al., 2010 |
| ***SUL*** | 0.11 | - | 1.52 | - | 0.21 | - | 4.35 | | - | 0.87 | - | 12.27 | | - | 1.71 | - | 35.19 | | - | - | - |
| ***HI*** |  | 0.01 |  | 0.11 |  | 0.02 |  | | 0.10 |  | 0.06 |  | | 0.89 |  | 0.14 |  | | 0.78 |  |  |

**Table S8.** Values for environmental risk assessment

| **Contaminant** | **MEC_SURF._**  **(ng/g)** | **MEC_DEPTH_ (ng/g)** | **Terrestrial organisms** | | | | | | | | | | | | **Aquatic organisms** | | | | | |
| --- | --- | --- | --- | --- | --- | --- | --- | --- | --- | --- | --- | --- | --- | --- | --- | --- | --- | --- | --- | --- |
|  |  |  | ***Earthworm*** | | | | ***Plant*** | | | | ***Fungi*** | | | |  |  |  |  |  |  |
|  |  |  | **PNEC_S_ (µg/kg)** | **RQ0-5** | **RQ25-30** | **Effect** | **PNEC_S_ (µg/kg)** | **RQ0-5** | **RQ25-30** | **Effect** | **PNEC_S_**  **(µg/kg)** | **RQ0-5** | **RQ25-30** | **Effect** | **PNEC_W_**  **(µg/L)** | **kd^a^ (L/kg)** | **PNEC_S_ (µg/kg)** | **RQ0-5** | **RQ25-30** | **Effect** |
| ***ACT*** | 10.60 | 11.60 | 662.1 | 0.02 | 0.02 | Mortality | - | - | - | - | - | - | - | - | 3.3 | 32 | 106 | 0.10 | 0.11 | Intoxication  Immobile |
| ***ATE*** | 63.68 | 19.05 | 1000 | 0.06 | 0.02 | Survival  Reproduction | 1000 | 0.06 | 0.02 | Emergence | - | - | - | - | 28 | 15 | 420 | 0.15 | 0.05 | Intoxication  Immobile |
| ***CAF*** | 588.34 | 425.63 | - | - | - | - | - | - | - | - | - | - | - | - | 0.44 | 25 | 11.0 | 53.5 | 38.7 | Feeding behavior |
| ***CBZ*** | 45.59 | 16.70 | - | - | - | - | 10 | 4.56 | 1.67 | Growth weight and lenght  Damage leaf  Mortality | 10 | 4.56 | 1.67 | Population abundance | 0.243 | 13 | 3.16 | 14.4 | 5.28 | Growth weight |
| ***DCF*** | 36.59 | 18.90 | 78.43 | 0.47 | 0.24 | Mortality | - | - | - | - | - | - | - | - | 2.919 | 9 | 26.3 | 1.39 | 0.72 | Mortality |
| ***ERY*** | 8.96 | 3.69 | - | - | - | - | - | - | - | - | - | - | - | - | 0.11 | 68 | 7.48 | 1.20 | 0.49 | Population abundance |
| ***IND*** | 46.04 | 50.83 | - | - | - | - | - | - | - | - | - | - | - | - | 12.59 | 32 | 403 | 0.11 | 0.13 | Intoxication immobile |
| ***KTP*** | 49.07 | 47.44 | - | - | - | - | - | - | - | - | - | - | - | - | - | 9 | - | - | - | - |
| ***NPX*** | 31.24 | 52.04 | - | - | - | - | - | - | - | - | - | - | - | - | 38.19 | 11 | 420 | 0.07 | 0.12 | Reproduction |
| ***SMX*** | 34.95 | 34.90 | 4000 | 0.01 | 0.01 | Mortality | 13 | 2.69 | 2.68 | Growth lenght |  | - | - |  | 0.14 | 8 | 1.12 | 31.2 | 31.2 | Population abundance |
| ***SUL*** | 337.84 | 281.03 | - | - | - | - | - | - | - | - | - | - | - | - | - | - | - | - | - | - |

^a^Barron et al., 2009; MEC_SURF._: maximum environmental concentration of each pharmaceutical detected on the surface of the soil; MEC_DEPTH_: maximum environmental concentration of each pharmaceutical detected on the depth of the soil; RQ0-5: risk quotient on the soil at 0-5 cm; RQ25-30: risk quotient on the soil at 25-30 cm; PNEC_S_: Predicted No-Effect Concentrations in soil; PNEC_W_: Predicted No-Effect Concentrations in water.

**REFERENCES**

Barron, L., Havel, J., Purcell, M., Szpak, M., Kelleher, B., & Paull, B. (2009). Predicting sorption of pharmaceuticals and personal care products onto soil and digested sludge using artificial neural networks. *Analyst*, *134*(4), 663-670. <https://pubs.rsc.org/en/content/articlehtml/2009/an/b817822d>

Bruce, G. M., Pleus, R. C., & Snyder, S. A. (2010). Toxicological relevance of pharmaceuticals in drinking water. *Environ. Sci. Technol., 44* (14), pp. 5619-5626, <https://doi.org/10.1021/es1004895>

Campagna-Fernandes, A. F., Marin, E. B., & Penha, T. H. F. L. (2016). Application of root growth endpoint in toxicity tests with lettuce (Lactuca sativa). *Ecotoxicology and Environmental Contamination*, *11*(1), 27-32. <https://doi.org/10.5132/eec.2016.01.05>

Nawrot, P., Jordan, S., Eastwood, J., Rotstein, J., Hugenholtz, A. & Feeley, M. (2003). Effects of caffeine on human health. Food Addit. Contam., 20 (1), pp. 1–30, <https://doi.org/10.1080/0265203021000007840>

Patel, M.; Kumar, R.; Kishor, K.; Mlsna, T.; Pittman, C. U., Jr; Mohan, D. Pharmaceuticals of Emerging Concern in Aquatic Systems: Chemistry, Occurrence, Effects, and Removal Methods. Chem. Rev. 2019, 119 (6), 3510– 3673, [https://doi.org/10.1021/acs.chemrev.8b00299](https://pubs.acs.org/doi/10.1021/acs.chemrev.8b00299)
